# Kinetic Modeling and Quantification of Pressure-Derived Oxygen Accumulation from Anthracene Endoperoxides under Confined Conditions

**DOI:** 10.1021/acsomega.6c06043

**Published:** 2026-08-06

**Authors:** Abhishek Sharma, Vanessa Barth, Chandrika Sethumadhavan, Frank Goldschmidtboeing, Henning J. Jessen, Laura M. Comella

**Affiliations:** † Cluster of Excellence livMatS at FIT−Freiburg Center for Interactive Materials and Bioinspired Technologies, University of Freiburg, Georges-Köhler-Allee 105, 79110 Freiburg, Germany; ‡ Laboratory for the Design of Microsystems, Department of Microsystems Engineering (IMTEK), University of Freiburg, Georges-Köhler-Allee 102, 79110 Freiburg, Germany; § Institute of Organic Chemistry, University of Freiburg, Albertstr. 21, 79104 Freiburg, Germany; ∥ Institute of Energy Efficient Mobility, 38992 Karlsruhe University of Applied Sciences, Moltkestr. 30, 76133 Karlsruhe, Germany

## Abstract

The controlled release of gas is central to chemically triggered systems and pressure-driven processes, where both the magnitude and temporal evolution of gas must be predictable. Anthracene-endoperoxides (ANT-EPO) are a class of oxygen-releasing molecules that decompose with well-defined stoichiometry and temperature-dependent kinetics, making them attractive candidates for tunable chemical sources. Prior work has demonstrated optical and pressure-based monitoring of ANT-EPO decomposition, providing valuable insights into its oxygen release behavior. The present work establishes a kinetic model to quantify and estimate oxygen release from ANT-EPO under confined geometries, using pressure as an experimental observable. Pressure–time data acquired using gauge and absolute sensors at ambient room temperature and 37 °C were converted into gas-phase oxygen accumulation using an ideal gas law formulation. The resulting profiles were described by first-order kinetics, yielding temperature-dependent rate constants that were independent of concentration. An apparent gas-phase recovery factor was introduced to account for nonideal conversion between the theoretical oxygen release capacity and the experimentally accessible oxygen amount recovered in the headspace. These parameters were integrated into a compact empirical-mechanistic model that relates concentration and temperature to pressure evolution within the defined chamber geometry. Model performance was evaluated through forward prediction of oxygen release using an independently measured concentration excluded from parameter extraction, demonstrating accurate prediction without refitting. This approach enables quantitative analysis and prediction of confined gas-evolving reactions and provides a basis for translating ANT-EPO reaction kinetics into pressure evolution under defined operating conditions. This work bridges responsive materials, chemical kinetics, and engineering applications, demonstrating ANT-EPO as a potential controllable oxygen source.

## Graphical abstract

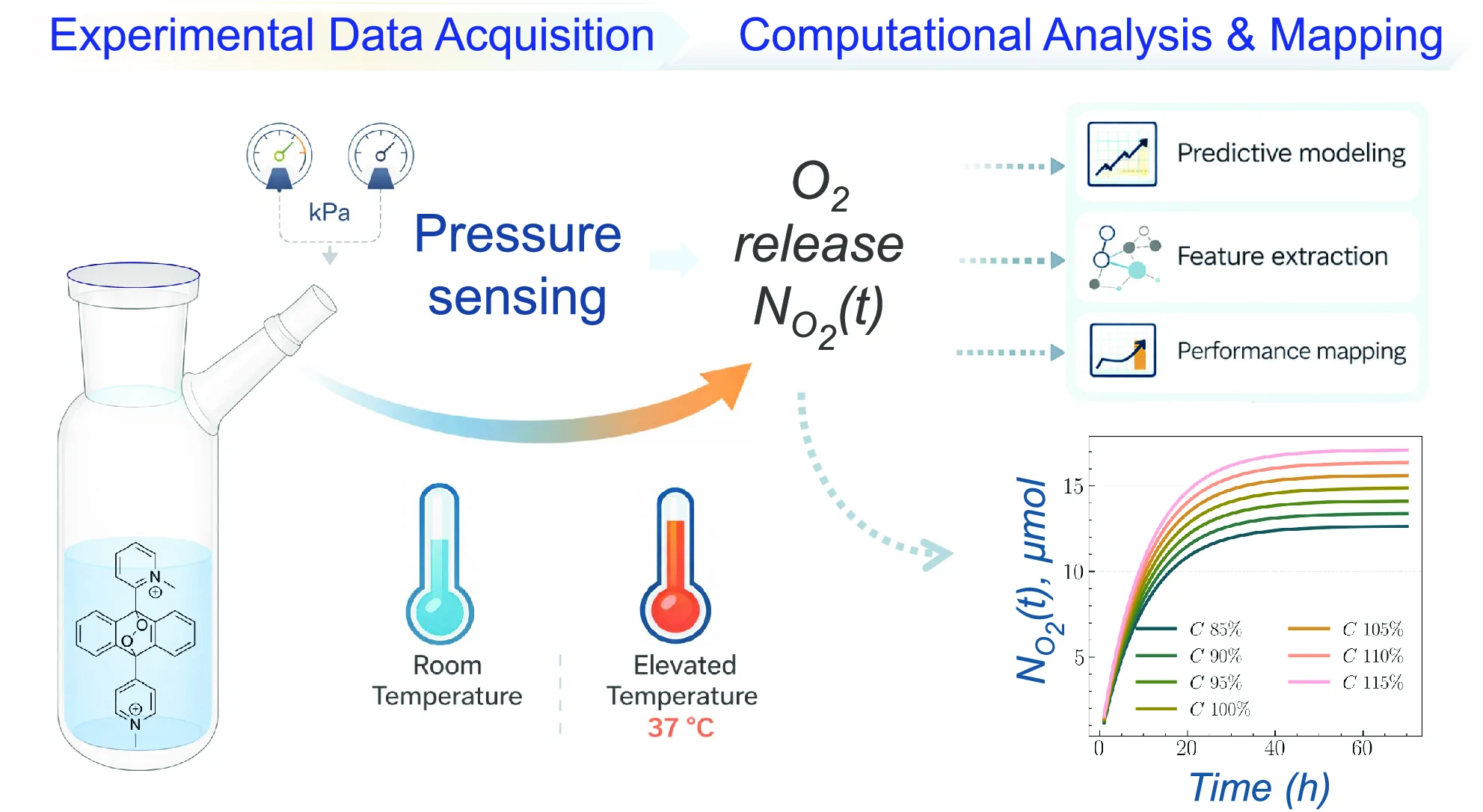

## 1 Introduction

The controlled generation of gases on demand is valuable to a wide range of sensing, actuation, and targeted gas delivery technologies.^1–3^ Pressure-driven microactuation, pneumatic control systems, and soft robotic components rely on predictable gas generation to achieve reproducible mechanical responses.^4–8^ For such systems, gas release must be quantifiable in both magnitude and time, so that the chamber geometry can be set with respect to the generated pressure and actuation thresholds.^1,9^ Several chemical reactions capable of producing gases are known, however, their practical use in confined systems is often limited by the lack of quantitative analysis that links chemical conversion to measurable pressure evolution.^10–12^

Anthracene endoperoxides (ANT-EPO) represent a distinct class of oxygen-storing molecules that undergo a clean cycloreversion reaction in aqueous environments.^13,14^ ANT-EPO dissolves in water and releases molecular oxygen with a well-defined 1:1 stoichiometry. The decomposition kinetics of ANT-EPO are temperature-dependent, allowing the rate of oxygen release to be tuned without altering the chemical composition. These properties make ANT-EPO attractive candidates for controlled oxygen delivery. Anthracene-derived endoperoxides have been exploited for singlet-oxygen detection^14–18^ and photodynamic therapy.^19–21^ Their photophysical properties and decomposition pathways are well characterized,^22,23^ yet few investigations have quantitatively linked ANT-EPO’s oxygen release kinetics to gas-phase accumulation under confined conditions.

Pressure-based responses arising from gas generation are well established across microfluidics, soft robotics, and responsive material systems.^3–5,24^ Pneumatic networks, phase change actuators, and gas-driven membrane systems have demonstrated that pressure in the order of tens of kilopascals is sufficient to induce deflections and fluid transport in compliant structures.^25–28^ A broad range of gas generation strategies has been investigated, including compressed air delivery, liquid–gas phase transitions, energetic material decomposition, and solvent sorption.^29,30^ Across these systems, predictive description of pressure evolution is recognized as a prerequisite for reliable process design, yet is often challenging due to multiphysics coupling, geometry dependence, and poorly characterized reaction kinetics.^31–33^

In the specific context of ANT-EPO, recent work has shown that their decomposition can be reliably tracked optically via absorbance-based detection of ANT-EPO and its product anthracene (ANT), providing indirect but real-time monitoring of oxygen release kinetics.^34,35^ Furthermore, pressure-based characterization systems have directly captured the headspace pressure generated during ANT-EPO decomposition in sealed chambers,^24,36^ confirming that oxygen release leads to measurable and reproducible pressure build-up. These studies have established the qualitative dependence of pressure evolution from oxygen release on concentration, solution-to-headspace ratio, and temperature.

Despite these advances, a critical gap remains between measured pressure signals and the actual amount of oxygen released into the gas phase.^37,38^ Pressure traces alone do not reveal how efficiently the overall oxygen release capacity of ANT-EPO is converted into measurable gas confined within the headspace. Moreover, a quantitative framework that provides a basis for extrapolating pressure evolution across concentrations and temperatures remains unexplored. Open questions include how much of the theoretical oxygen capacity is recovered as measurable gas,^31^ how the pressure-derived gas-phase recovery varies with temperature and measurement geometry, and under what conditions the classical kinetic description of ANT-EPO remains physically valid. Without a quantitative model linking pressure, oxygen release, and kinetic parameters, it remains difficult to determine whether a given amount of ANT-EPO is suitable for target pressure generation or how chamber dimensions should be selected to achieve a desired pressure profile.

The present work addresses this gap by establishing a kinetic model for oxygen release from ANT-EPO under confined volumes, using pressure as an observable. Pressure–time data obtained from ANT-EPO solutions at multiple concentrations and two temperature conditions at room temperature (RT) and 37 °C were converted into gas-phase oxygen accumulation using an ideal gas law formulation. The proposed framework (i) establishes a quantitative link between pressure evolution and gas-phase oxygen accumulation, (ii) extracts temperature-dependent rate constants using first-order kinetics applied to gas-phase oxygen accumulation profiles, and (iii) defines an apparent gas-phase recovery factor that scales the theoretical release capacity to the recovered gas-phase amount within the analysis window. These experimentally derived parameters were integrated into a compact empirical-mechanistic model enabling prediction of oxygen release for specified concentrations and operating temperatures within the defined chamber geometry. Model performance was evaluated using an independently measured ANT-EPO concentration that was excluded from parameter extraction, providing forward validation without refitting. To the knowledge of the authors, no integrated approach exists to assess ANT-EPO decomposition kinetics with gas-phase oxygen accumulation under varying environmental conditions, which provides an understanding and practical utilization of these oxygen releasing compounds.

Through these contributions, this work defines a quantitative framework for assessing the operating range of ANT-EPO as an oxygen-releasing source, through pressure as a direct observable. The resulting approach provides a pathway for translating controlled oxygen release into pressure evolution under defined operating conditions and provides a basis for incorporating oxygen-releasing reactions into responsive material systems and chemically driven components.

## 2 Materials and Methods

### 2.1 Chemicals and Reagents

Oxygen-release from anthracene-endoperoxide (ANT-EPO) was characterized through long-duration measurements in aqueous solution. The decomposition of ANT-EPO follows a 1:1 stoichiometry, forming anthracene (ANT) and releasing molecular oxygen into the surrounding environment.^13^ This study focuses on the forward decomposition process of ANT-EPO, with characteristic half-lives of 35.8 h at room temperature (RT) and 4.7 h at 37 °C (Figure 1(a)).^13,24^ Under sealed conditions, the released oxygen accumulates in the headspace and leads to a measurable pressure increase that provides a direct physical measure of gas-phase oxygen accumulation.^24,36^

**Figure 1:**
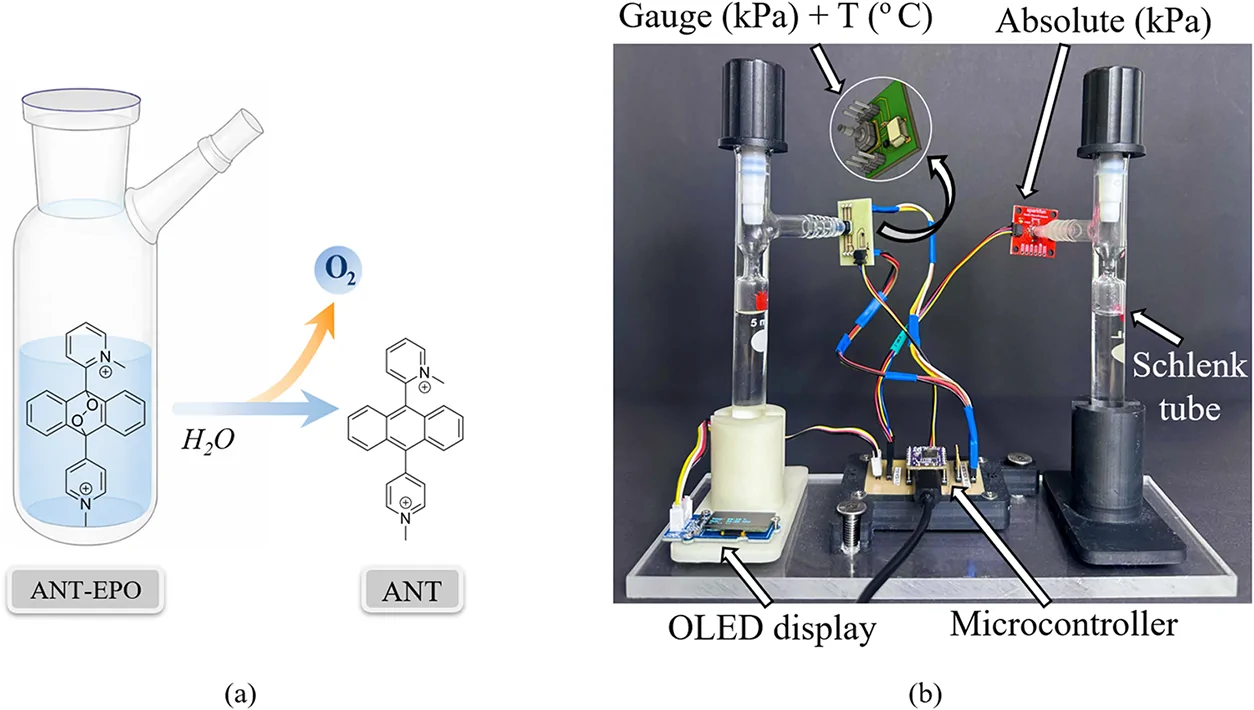
(a) Decomposition of anthracene-endoperoxide (ANT-EPO) in aqueous solution, releasing molecular oxygen and forming anthracene (ANT). The setup records the pressure generated from the oxygen release during the forward reaction. (b) Experimental setup for pressure-based characterization of oxygen release under confined conditions. Two 10 mL Schlenk tubes containing ANT-EPO solutions are equipped with gauge and absolute pressure sensors, as well as a temperature sensor. The sensing module interfaces with a low-power microcontroller for continuous real-time data acquisition.

ANT-EPO solutions were prepared in deionized (DI) water at discrete concentrations *M*_1_–*M*_4_, as summarized in Table 1. For measurements conducted at 37 °C, concentrations *M*_1_–*M*_3_ were characterized using both gauge and absolute pressure sensors. At RT, concentrations *M*_1_, *M*_2_ and *M*_4_ were measured using the gauge pressure sensor, with additional absolute-pressure data acquired for *M*_1_. The concentration range was selected to balance measurable pressure generation against long-duration observability. At lower concentrations, the pressure signal approaches the noise floor of extended measurements, while higher concentrations lead to rapid saturation and increased sensitivity to nonideal gas-phase recovery effects.^18,24^ The chosen concentrations therefore ensure resolvable kinetics and well-defined high-recovery behavior within experimentally accessible time scales.

**Table 1:** Anthracene-Endoperoxide (ANT-EPO) Concentrations Investigated and Corresponding Temperature-Resolved Datasets^a^

sample	[ANT-EPO] mM	RT data sets	37 °C data sets
*M*_1_	1.2	gauge, absolute	gauge, absolute
*M*_2_	1.6	gauge	gauge, absolute
*M*_3_	2.0		gauge, absolute
*M*_4_	2.5	gauge	

a Each sample (*n* = 1) was divided into four Schlenk tubes to allow parallel measurements at the room temperature (RT) setup and at 37 °C using gauge and absolute pressure sensors. The table reflects the available experimental datasets for each concentration-sensor-temperature configuration.

All experiments were performed using a fixed solution volume of 6 mL and a headspace volume of 4 mL in 10 mL Schlenk tubes, maintaining a constant solution-to-headspace ratio across all conditions. This geometry was intentionally kept constant across all conditions to ensure reproducibility and direct comparison of reaction kinetics. Two identical experimental setups were operated in parallel at RT and at 37 °C in a controlled thermal environment, as shown in Figure 1(b).

### 2.2 Experimental Setup

Each experimental system comprised of two sealed Schlenk tubes connected to a compact sensing module (Figure 1(b)). One tube was equipped with a gauge pressure sensor (ABP2LANT160MGSA3, 16 kPa, Honeywell) and a temperature–humidity sensor (SHT45, Sensirion), while the second tube was equipped with an absolute pressure sensor (MPRLS0025PA00001A, 25 psi, Honeywell). The temperature sensor was mounted adjacent to the gauge pressure sensor and measured the internal air temperature of the sealed module near the Schlenk tube. Given the small solution volume, thin glass wall, and long experimental time scales, thermal equilibrium between solution, headspace, and internal air was assumed. Both tubes were filled with identical ANT-EPO solutions to ensure that gauge and absolute measurements reflected the same process under identical geometry and thermal conditions. The gauge sensor, referenced to ambient atmosphere, provided high sensitivity in the low-pressure range and was effective for detecting subtle kinetic transitions. The absolute sensor, referenced to vacuum, provided pressure readings independent of ambient fluctuations. Comparing the signals from both sensors enabled reliable separation of reaction-driven pressure changes from environmental variations and served as an internal consistency check.^39^ Relative humidity was monitored to assess environmental stability but was not included as a model parameter, as its contribution to the measured pressure remains constant under sealed conditions.

Signals were recorded continuously at 1 Hz using a low-power microcontroller (QT Py ESP32, Adafruit). Individual measurements extended beyond 70 h at 37 °C and over 200 h at RT, capturing the main kinetic transients and the high-recovery region used for pressure-derived gas-phase parametrization. For RT experiments, ambient temperature varied depending on season and measurement period. Mean temperature, standard deviation, and minimum/maximum values for each data set are summarized in Table S1 (Supporting Information). Raw pressure data were preprocessed using an established signal-processing pipeline consisting of baseline correction, adaptive Savitzky-Golay (SG) smoothing, and discrete wavelet transform (DWT) denoising for the respective data set.^24,36,40,41^ This procedure removed low-frequency drift and high-frequency noise while preserving the underlying kinetic trend.

The system geometry, headspace-to-solution ratio, and sensor configuration were kept constant throughout all experiments to ensure reproducibility and direct comparison across concentrations, temperatures, and sensing modalities.

### 2.3 Physical Model: Gas-Phase Oxygen Accumulation

Measured pressure–time signals, *P*(*t*), were converted into the corresponding gas-phase oxygen accumulation using the ideal gas law.^42^ For each experiment, the amount of oxygen accumulated in the headspace was calculated as

1 $$N_{\mathrm{gas},\exp}\left(t\right)=\frac{\left(P\left(t\right)-P_{\mathrm{init}}\right)V_{g}}{\mathrm{RT}\left(t\right)}$$

where *P*_init_ is the initial measured headspace pressure at the start of the experiment, *V*_g_ is the headspace volume, *T*(*t*) is the measured temperature, and *R* is the universal gas constant. The resulting gas-phase oxygen accumulation, *N*_gas,exp_(*t*), is independent of pressure sensor type, which enables direct comparison between gauge and absolute pressure measurements.

The time evolution of gas-phase oxygen accumulation was described using a first-order kinetic model^43^

2 $$N_{\mathrm{gas}}\left(t\right)=N_{0}\left(1-\exp^{-\mathrm{kt}}\right)$$

where *k* is the first-order rate constant and *N*_0_ is the apparent recovered gas-phase oxygen amount.

Based on the reported 1:1 stoichiometry of ANT-EPO decomposition,^13^ the theoretical maximum releasable oxygen is given by the product of ANT-EPO concentration, *C*, and the solution volume, *V*_sol_. Experimentally, the pressure-derived recovered gas-phase amount can be lower than the theoretical oxygen release capacity because the measured pressure reflects the gas-phase oxygen accessible to the headspace. This may also include nonideal effects such as partial dissolution of oxygen in the liquid phase, incomplete conversion or minor system losses. To account for these effects, an apparent gas-phase recovery factor η was defined as

3 $$\eta =\frac{N_{0}}{CV_{\mathrm{sol}}}$$

The apparent gas-phase recovery factor η is dimensionless and scales the total amount of oxygen transferred into the gas-phase. It does not influence the reaction kinetics and affects only the magnitude of the gas-phase oxygen yield. Using this definition, the recovered gas-phase model yield can be expressed as

4 $$N_{0}=\eta CV_{\mathrm{sol}}$$

The gas-phase is treated as ideal, the headspace volume is taken as fixed, the liquid volume is assumed constant during the experiment, and the measured internal air temperature is used as a representative temperature for the headspace. These assumptions are appropriate for the low-pressure range and fixed Schlenk-tube geometry used in this study. However, they introduce uncertainty through pressure-sensor calibration, filling-volume tolerances, residual temperature gradients, and oxygen partitioning between the liquid and gas phases. The apparent gas-phase recovery factor η should therefore not be interpreted as a universal molecular yield of ANT-EPO. Instead, η is a setup-specific factor that describes the fraction of theoretically releasable oxygen recovered as measurable gas-phase oxygen in the present solution-to-headspace configuration and within the analyzed time window. Changing the total confinement volume or the liquid-to-headspace ratio would alter gas–liquid partitioning and can therefore change η. Extrapolation to substantially different confinement geometries would require redetermination of η or an explicit gas–liquid partitioning model.

Substituting eq 4 into eq 2 provides the modeled gas-phase oxygen accumulation

5 $$N_{\mathrm{gas},\mathrm{mod}\mathrm{el}}\left(t\right)=\eta CV_{\mathrm{sol}}\left(1-\exp^{-k\left(T\right)t}\right)$$

Here, the explicit temperature dependence of the rate constant, *k*(*T*), is indicated. The corresponding pressure evolution can be obtained by rearranging the ideal gas law

6 $$P_{\mathrm{model}}\left(t\right)=\frac{N_{\mathrm{gas},\mathrm{mod}\mathrm{el}}\left(t\right)\mathrm{RT}}{V_{g}}$$

eqs 5 and ([Disp-formula eq6]) together define a kinetic framework capable of predicting both the time-dependent oxygen evolution and the maximum pressure output for specified concentrations and temperatures within the defined fixed-volume geometry.

### 2.4 Parameter Extraction and Validation

For each experimental condition, the gas-phase oxygen accumulation profiles *N*_gas_ (*t*), calculated using eq 1 were fitted directly to the first-order kinetic model (eq 2), yielding the fitted parameters *N*_0,exp_ and *k*_exp_.

For RT measurements, where small but finite temperature fluctuations occurred over multiday experiments, Arrhenius-based fitting was applied using the time-resolved temperature signal to account for temperature dependence of ANT-EPO decomposition kinetics. To obtain the temperature-specific kinetic parameters, fitted rate constants, *k*_exp_, from experiments performed at the same temperature were pooled across concentrations and pressure sensor types. A representative rate constant *k*(*T*) was determined as the mean value with associated uncertainty, reflecting the interpretation that ANT-EPO decomposition kinetics are governed primarily by temperature.^13,24^ Apparent gas-phase recovery factors η, were obtained for each experiment using eq 3, and representative η­(*T*) values were obtained by averaging across concentrations–sensor data sets for each temperature condition.

The final model (eq 5) requires as inputs only the initial concentration, *C*, solution and headspace volumes, *V*_sol_, and *V*_g_, temperature, *T*, and the experimentally determined parameters *k*(*T*) and η­(*T*). Pressure–time data were used exclusively for parameter extraction and validation, and were not required as inputs during prediction.

To assess predictive capability, an independent ANT-EPO concentration was prepared and measured under identical geometric and thermal conditions but was excluded from parameter extraction. Pressure–time data from this validation experiment were processed using the same analysis pipeline, but were not used to update or refit any model parameters. Model predictions were generated using the previously determined *k*(*T*) and η­(*T*) values and compared directly against the experimental measurements. No parameters were refitted for the validation data set.

## 3 Results and Discussion

### 3.1 Pressure-Derived Gas-Phase Oxygen Accumulation

Pressure–time measurements obtained from gauge and absolute sensors were converted into gas-phase oxygen accumulation, *N*_gas,exp_(*t*), using the ideal gas law (eq 1, Section 2.3). This transformation yields a physically consistent representation of oxygen release that is directly comparable across pressure referencing schemes and forms the basis for all subsequent kinetic analysis and modeling.

Figure 2 shows representative *N*_gas,exp_(*t*) profiles for ANT-EPO sample *M*_1_ obtained from gauge and absolute pressure measurements at RT and 37 °C. For each temperature condition, *N*_gas,exp_(*t*) profiles reconstructed independently from both sensors exhibit similar trajectories over time. This agreement indicates that differences in sensor referencing do not influence the gas-phase oxygen accumulation. First-order kinetic fits were overlaid to illustrate the smooth temporal evolution and well-defined accumulation behavior of the oxygen release profiles.

**Figure 2:**
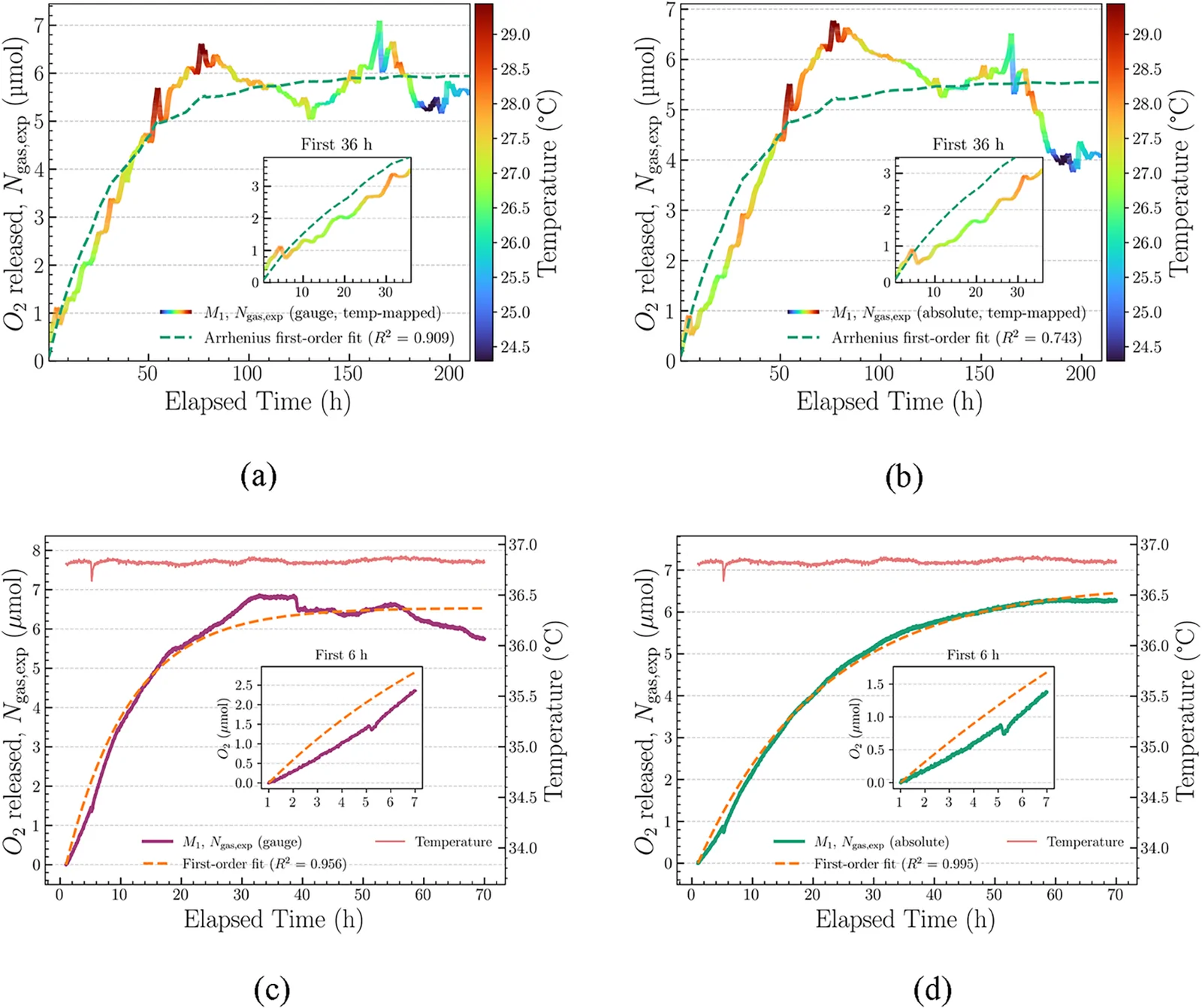
Gas-phase oxygen accumulation derived from pressure measurements for ANT-EPO sample M_1_ and fitted with the first-order kinetic model. (a) *N*_gas,exp_(*t*) obtained from gauge pressure measurements at room temperature (RT). (b) *N*_gas,exp_(*t*) obtained from absolute pressure measurements at RT. In panels (a) and (b), the color scale represents the measured internal module temperature at each time point and visualizes minor ambient temperature fluctuations during the long-duration RT measurements. (c) *N*_gas,exp_(*t*) from gauge pressure measurements at 37 °C. (d) *N*_gas,exp_(*t*) from absolute pressure measurements at 37 °C. Insets highlight the early time regime of oxygen accumulation at the start of the reaction.

At RT, the color of each data point in Figure 2a,b represents the measured internal temperature at the corresponding measurement time. The temperature is encoded as a colormap along the gas-phase oxygen accumulation curve. This visualization was included because the RT measurements extended over more than 200 h and were subject to small ambient temperature fluctuations (Table S1, Supporting Information). Warmer intervals correspond to slightly faster local oxygen accumulation, whereas cooler intervals coincide with slightly reduced slopes. These variations are consistent with the known temperature sensitivity of ANT-EPO decomposition kinetics.^24,36^ At 37 °C, measurements were performed under controlled thermal conditions, resulting in smoother kinetic profiles without comparable ambient-temperature influence.

Measurements at 37 °C span more than 70 h, while RT experiments extend beyond 200 h, allowing the main kinetic transients and high recovery regime to be captured without extrapolation. Based on reported half-lives, more than 98% of the initially present ANT-EPO was expected to decompose over these respective time scales. The pressure-derived profiles therefore provide sufficient temporal coverage to extract effective kinetic parameters and representative gas-phase recovery factors.

Sample *M*_1_ is shown as a representative case and complete data sets are available for both pressure sensor types and temperature conditions, enabling a direct and internally consistent comparison. The consistency observed in Figure 2 establishes *N*_gas,exp_(*t*) as a physically meaningful kinetic variable suitable for quantitative modeling. Corresponding *N*_gas,exp_(*t*) profiles for all concentrations and experimental configurations are provided in the Supporting Information (Figures S1–S6). Additionally, log–log comparisons between gas-phase oxygen accumulation derived from gauge and absolute pressure sensing are also provided in the Supporting Information (Figures S7–S10), demonstrating consistent scaling and preservation of release trends across concentrations and temperatures. This provides a robust foundation for kinetic parameter extraction, which is addressed in the following section.

### 3.2 Kinetic Parameters Extraction

All *N*_gas,exp_(*t*) profiles were analyzed using the first-order kinetic model described in eq 2 (Section 2.3). As shown in Section 3.1, gas-phase oxygen accumulation reconstructed from different pressure referencing schemes was consistent after conversion and was therefore treated equivalently during kinetic analysis.

First-order fits to *N*_gas,exp_(*t*) at RT and 37 °C show strong agreement across all investigated concentrations listed in Table 1, capturing both the initial rise in oxygen accumulation and the approach to saturation. A pronounced acceleration of oxygen release kinetics is observed at 37 °C relative to RT, consistent with thermally activated ANT-EPO decomposition.

Effective rate constants *k*_exp_ extracted from all measurements are summarized in Table S2 (Supporting Information). Under ambient RT conditions, Arrhenius-based fitting was applied using the time-resolved temperature signal to account for measured temperature fluctuations (Section 2.4). This approach explicitly accounts for transient thermal variations rather than treating RT measurements as strictly isothermal. Under controlled conditions at 37 °C, a single characteristic rate constant was extracted for each measurement.

For a given temperature, *k*_exp_ values exhibit only minor variation across the investigated ANT-EPO concentration range, with no systematic concentration dependence within experimental uncertainty. This behavior indicates that the observed kinetics are governed primarily by temperature rather than concentration and measurement modality over the investigated range. Rate constants were therefore pooled across concentrations and pressure sensor types to obtain representative temperature-specific values *k*(*T*).

Averaging across all Arrhenius-based RT measurements yields an effective rate constant of *k*_RT,gauge_ = (2.04 ± 1.11) × 10^–2^ h^–1^, where the standard deviation reflects ambient thermal variability during the experiments. At 37 °C, pooled rate constants *k*_37,gauge_ = (9.75 ± 1.75) × 10^–2^ h^–1^ and *k*_37,absolute_ = (5.74 ± 1.83) × 10^–2^ h^–1^, were obtained. The difference was treated as sensor configuration specific uncertainty in the pressure-derived kinetic estimate rather than concentration dependent decomposition kinetics. These pooled values were used as fixed kinetic inputs for all subsequent predictive modeling, enabling a compact description of ANT-EPO oxygen release kinetics with minimal parameter redundancy.

### 3.3 Apparent Gas-Phase Oxygen Recovery

The first-order kinetic fitting provided the magnitude term as described in eq 3 (Section 2.3), was expressed relative to the theoretical oxygen capacity expected from ANT-EPO decomposition. The oxygen recovered in the headspace provides an apparent gas-phase recovery factor, η, as the ratio between the experimentally observed gas-phase oxygen amount and the theoretical release capacity.

Extracted recovery factors are summarized in Table S3 (Supporting Information). Across the investigated ANT-EPO concentration range, η remains bounded and exhibits only moderate variation, indicating that gas-phase oxygen yield is largely independent of concentration for a fixed chamber geometry. At RT, long-duration traces can exhibit late-time pressure relaxation and thermal drift after the main release phase, therefore eta was interpreted as a representative gas-phase recovery factor within the analysis window rather than as a final thermodynamic plateau.

Recovery factors derived from the available pressure referencing schemes were pooled by temperature to obtain representative η­(*T*) values. Averaging across the RT data sets yielded η_RT_ = (93.1 ± 1.2) %, while at 37 °C data sets yielded η_37_ = (92.87 ± 3.76) %. The recovery factors reported here are therefore used as geometry-specific apparent recovery parameters for forward validation in the same fixed confinement geometry. They should not be interpreted as geometry-independent thermodynamic constants. Their main purpose is to translate the theoretical ANT-EPO oxygen capacity into the experimentally accessible gas-phase oxygen amount for the defined headspace-to-solution configuration. The extracted rate constant *k*(*T*) describes the temporal release behavior, whereas η scales the recovered gas-phase magnitude.

Together with the temperature-dependent rate constants *k*(*T*) identified in Section 3.2, these recovery factors provide the compact parameter set required for the kinetic model. This parametrization enables prediction of gas-phase oxygen accumulation and corresponding pressure evolution under defined operating conditions as outlined in Section 2.4.

### 3.4 Model Validation

The predictive capability of the proposed kinetic framework was evaluated using an independently prepared ANT-EPO solution with a concentration of 2.67 mM, which was excluded from parameter extraction. Model predictions were generated in a forward-prediction manner using fixed *k*(*T*) and η­(*T*) values obtained from the training data sets, without refitting or parameter adjustment.

Figure 3 presents the forward validation results at RT. Panel (a) compares experimentally derived *N*_gas,exp_(*t*), obtained from gauge pressure measurements, with the corresponding model prediction. Under ambient RT conditions, the model captures the experimentally observed gas-phase recovery magnitude, while deviations in early time slopes arise from sensitivity to small temperature fluctuations over long experimental durations.

**Figure 3:**
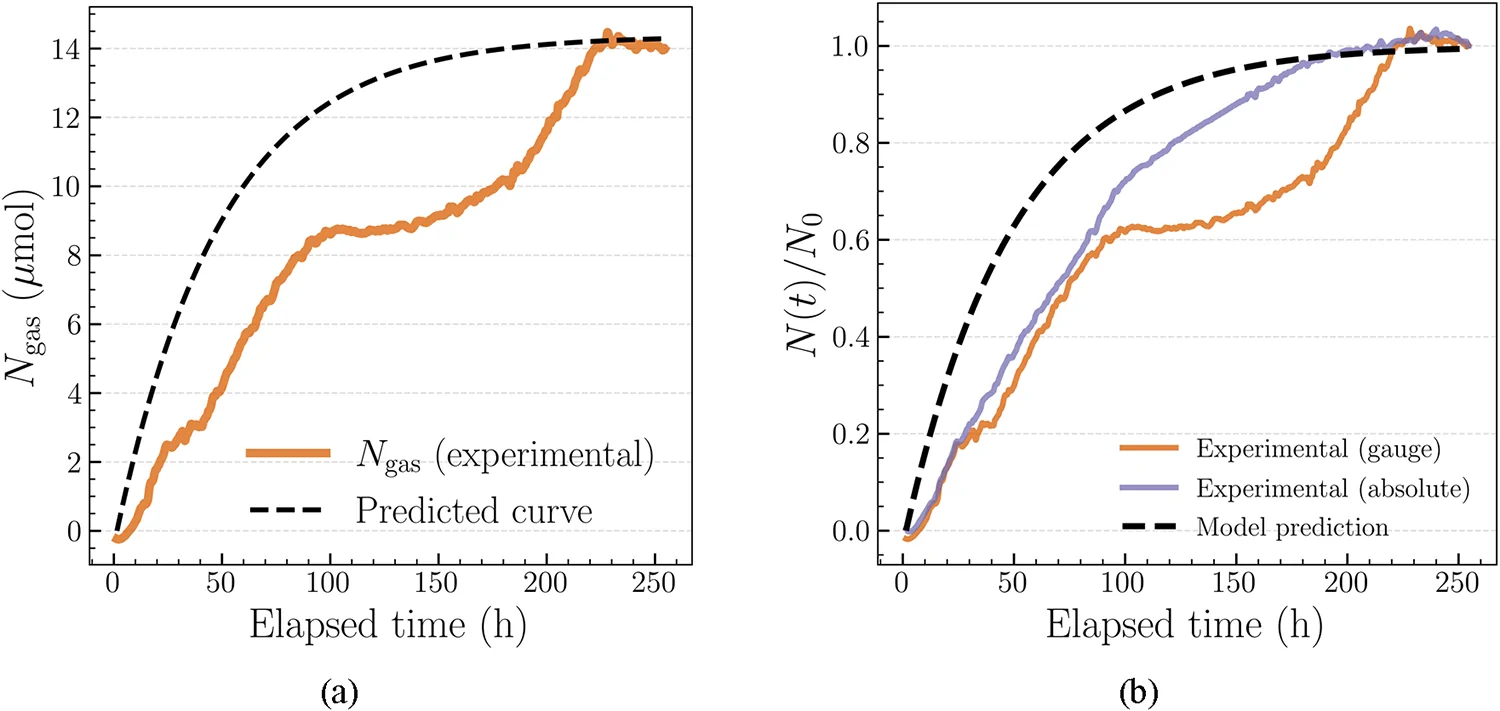
Forward model validation at room temperature (RT) using an unseen ANT-EPO concentration of 2.67 mM. (a) Gas-phase oxygen accumulation *N*_gas,exp_(*t*) derived from gauge pressure measurements (solid line, orange) compared with the model prediction (dashed line, black). Rate constants were obtained using Arrhenius-based fitting to account for ambient temperature variations, and all model parameters were fixed prior to validation. (b) Normalized oxygen accumulation *N*_gas,exp_(*t*)/*N*_0_ for gauge-derived data, absolute-derived data, and model prediction.

RT measurements spanned extended durations (>200 h) and were subject to slight ambient temperature variations (typically ±1–3 °C depending on measurement period; see Table S1, Supporting Information), which primarily influence early time kinetics rather than the recovered gas-phase magnitude. Panel (b) shows normalized oxygen accumulation profiles for gauge-derived data, absolute-derived data, and model prediction, normalized with respect to the recovered gas-phase oxygen amount. The close overlap of normalized curves indicates preservation of the overall kinetic shape under nonisothermal RT conditions and supports the applicability of the temperature-dependent kinetic formulation at ambient temperature.

Figure 4 shows the corresponding validation at 37 °C. The model accurately reproduces both the temporal evolution and the gas-phase recovery magnitude without systematic deviation. Normalized profiles again exhibit close overlap between experiment and model, demonstrating that the predicted kinetics are independent of sensor type and absolute scaling. Considering faster ANT-EPO’s reaction kinetics at 37 °C, characteristic release times defined as the time to reach 25% (*t*_25_) and 50% (*t*_50_) of the final oxygen yield provide a meaningful quantitative comparison in this regime and are summarized in Table S4 (Supporting Information). Absolute pressure validation results for both temperatures are provided in the Supporting Information (Figures S11 and S12).

Quantitative validation metrics, including root-mean-square error (RMSE), normalized RMSE, and Pearson correlation coefficients are summarized in Tables S5–S7 (Supporting Information). At RT, correlation coefficients remained high (*r* > 0.91), indicating preservation of overall release behavior despite slower kinetics and increased thermal variability. At 37 °C, strong linear agreement between predicted and experimental trajectories was observed (*r* > 0.98). Uncertainty propagation analyses based on experimentally observed variability in *k*(*T*) and η­(*T*), as well as sensitivity to concentration uncertainty (*C* ± 15%), are provided in Figures S13–S18 (Supporting Information). These analyses show that parameter variability primarily affects the gas-phase recovery magnitude, while the normalized kinetic shape was preserved. In all cases, experimental trajectories fall within the expected parameter bounds, and the model response scales linearly with concentration.

The closer agreement observed at 37 °C can be attributed to the more controlled thermal boundary conditions and the faster decomposition kinetics at this temperature. Under controlled 37 °C conditions, the reaction proceeds over a shorter effective time scale and is less affected by long-term ambient drift or low-frequency temperature fluctuations. In contrast, RT measurements extend over more than 200 h and are subject to small ambient temperature variations, which influence the local release rate and primarily affect the early time slope of the gas-phase oxygen accumulation profile. Therefore, the RT validation shows slightly larger deviations despite preserving the overall kinetic trend and recovered gas-phase magnitude. The improved agreement at 37 °C does not result from additional parameter adjustment, since the validation was generated without refitting.

Together, these results demonstrate that the proposed framework provides predictive kinetic descriptions of ANT-EPO oxygen release. By reproducing pressure-derived oxygen accumulation kinetics and recovered gas-phase magnitudes for an unseen concentration without refitting, the model establishes a quantitative link between ANT-EPO concentration, temperature, and pressure evolution under confined conditions.

**Figure 4:**
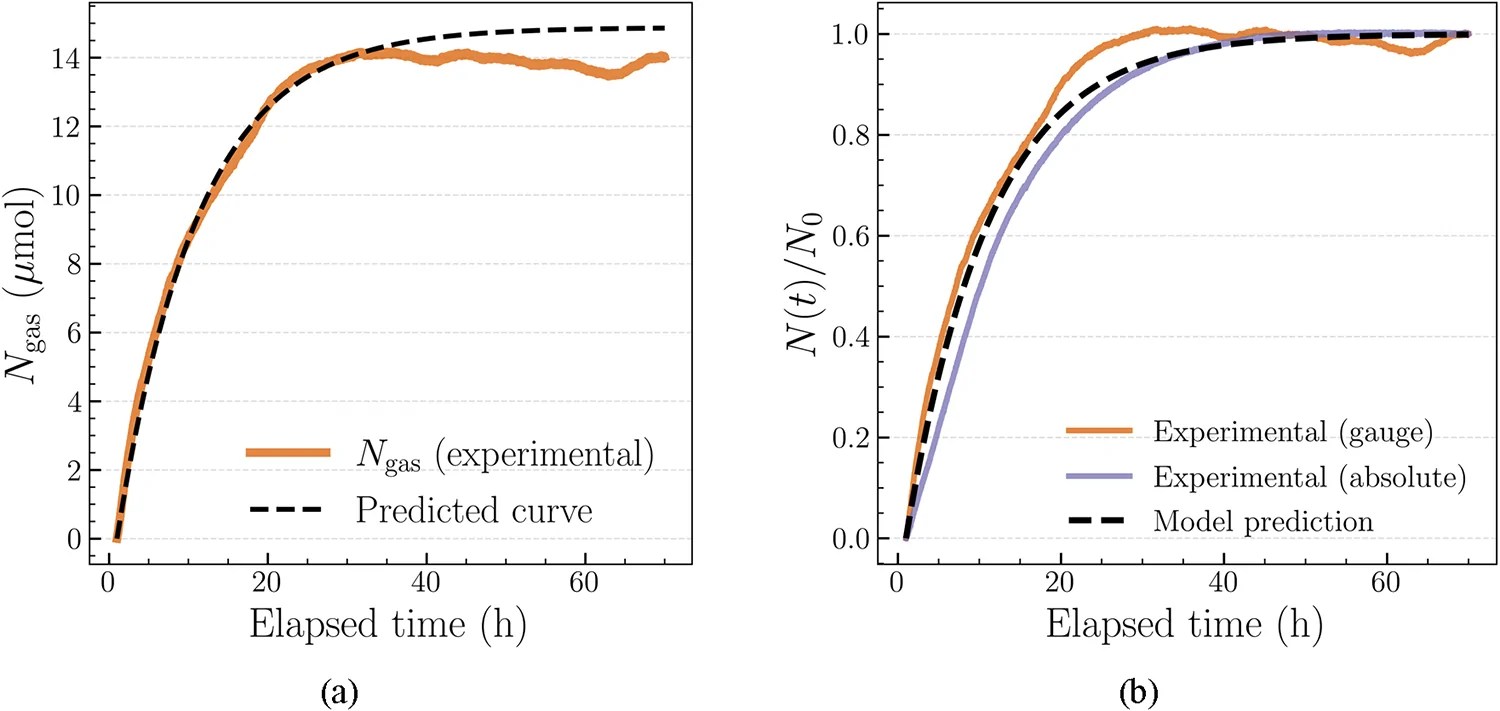
Forward model validation at 37 °C using an unseen ANT-EPO concentration of 2.67 mM. (a) Gas-phase oxygen accumulation *N*_gas,exp_(*t*) derived from gauge pressure measurements (solid line, orange) compared with the model prediction (dashed line, black). Model parameters *k*(37) and η(37) were fixed to values extracted from the training data set and were not refitted for this concentration. (b) Normalized oxygen accumulation *N*_gas,exp_(*t*)/*N*_0_ for gauge-derived data, absolute-derived data, and model prediction.

## 4 Conclusions

This work establishes a quantitative kinetic modeling framework for oxygen release from anthracene-endoperoxides (ANT-EPO) under confined conditions, using pressure as an experimental observable to reconstruct gas-phase oxygen accumulation. By converting pressure–time data into gas-phase oxygen accumulation, a sensor-independent representation of oxygen release was obtained, enabling consistent analysis across temperatures and concentrations. Within the investigated range, oxygen release kinetics were well described by a first-order model, with rate constants governed primarily by temperature. Beyond kinetic description, an apparent gas-phase recovery factor was introduced to account for nonideal conversion between the theoretical oxygen release capacity and the experimentally accessible gas-phase oxygen amount. The bounded and reproducible nature of this gas-phase recovery parameter separates intrinsic reaction kinetics from system-level recovery effects associated with the fixed chamber geometry and pressure measurements. Together, the temperature-dependent rate constant and recovery factor form a compact parameter set that enables quantitative prediction of gas-phase oxygen accumulation within the fixed-volume configuration investigated here. The predictive capability of the framework was demonstrated through forward validation using an independently prepared ANT-EPO concentration that was excluded from parameter extraction. The model accurately reproduced both the temporal evolution and the recovered gas-phase oxygen magnitude without refitting, confirming transferability of the extracted parameters under defined operating conditions. From a design perspective, the presented approach provides direct guidance for oxygen-driven pressure generation. This framework supports systematic analysis and comparison of confined gas-evolving reactions and offers a quantitative foundation for integrating oxygen-releasing kinetics into engineered systems where controlled pressure generation is required. The present model is directly applicable to fixed-volume systems, where the headspace volume is constant during oxygen release. A relevant extension of this framework is variable-volume systems, such as a balloon, flexible reservoir, or deflectable membrane coupled to the gas-evolving chamber. In such systems, released oxygen would contribute to both pressure build-up and to volume expansion or membrane deflection. In compliant confinement, ANT-EPO decomposition kinetics could still serve as the oxygen-generation source term, but the pressure estimation would require coupling the kinetic model to a time-dependent headspace volume or to a mechanical pressure–volume relation of the compliant element. Coupling the present kinetic framework with mechanical compliance therefore represents an important next step toward chemically driven actuation systems.

## Supplementary Material



## Notes

Additional experimental profiles (Figures S1–S10); validation plots (Figures S11 and S12); uncertainty propagation (Figures S13–S16); sensitivity analysis (Figures S17 and S18); temperature statistics (Table S1); kinetic parameters (Table S2); recovery factors (Table S3); and validation metrics (Table S4–S7) (PDF)
