# Supplementary material for: Kinetic Modeling and Quantification of Pressure-Derived Oxygen Accumulation from Anthracene Endoperoxides under Confined Conditions
Source: ACS Omega. 2026 Aug 6;11(32):48574–82. doi: 10.1021/acsomega.6c06043 (PMC13491977; doi:10.1021/acsomega.6c06043)
Supplement: Supplementary file 1 [file ao6c06043_si_001.pdf]

SUPPORTING INFORMATION

Kinetic Modeling and Quantification of Pressure-Derived Oxygen Accumulation from Anthracene Endoperoxides under Confined Conditions

Abhishek Sharma[a,b,*], *Vanessa Barth*[a,c], *Chandrika Sethumadhavan*[b], *Frank Goldschmidtboeing*[b], *Henning J. Jessen*[a,c], *Laura M. Comella*[a,d]

[a] Cluster of Excellence livMatS @ FIT-Freiburg Center for Interactive Materials and Bioinspired Technologies, University of Freiburg, Georges-Köhler-Allee 105, 79110 Freiburg, Germany

[b] Laboratory for the Design of Microsystems, Department of Microsystems Engineering (IMTEK), University of Freiburg, Georges-Köhler-Allee 102, 79110 Freiburg, Germany

[c] Institute of Organic Chemistry, University of Freiburg, Albertstr. 21, 79104 Freiburg, Germany

[d] Institute of Energy Efficient Mobility, Karlsruhe University of Applied Sciences, Moltkestr. 30, 76133 Karlsruhe, Germany

*Corresponding author: abhishek.sharma@livmats.uni-freiburg.de

1.1 Experimental Datasets and Raw Kinetics

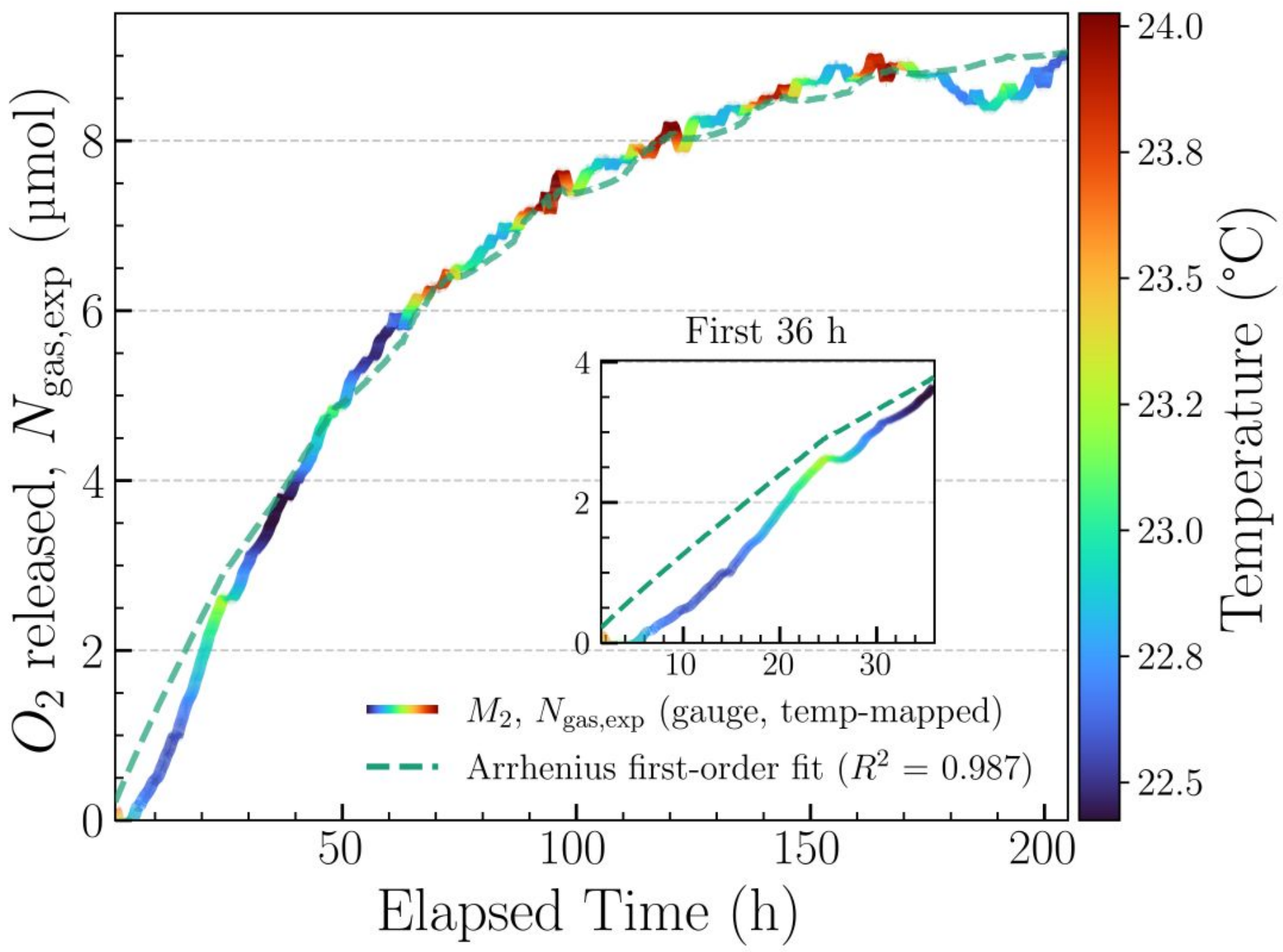

Figure S1: Oxygen release profile for sample M_2 (1.6 mM) measured at room temperature (RT) using gauge pressure sensing. The experimental gas-phase oxygen evolution, $N_{gas}(t)$, is shown as a function of elapsed time. Data illustrate slow-release kinetics under ambient conditions with temperature coded colormaps illustrating thermal drift effects characteristic of non-isothermal measurements.

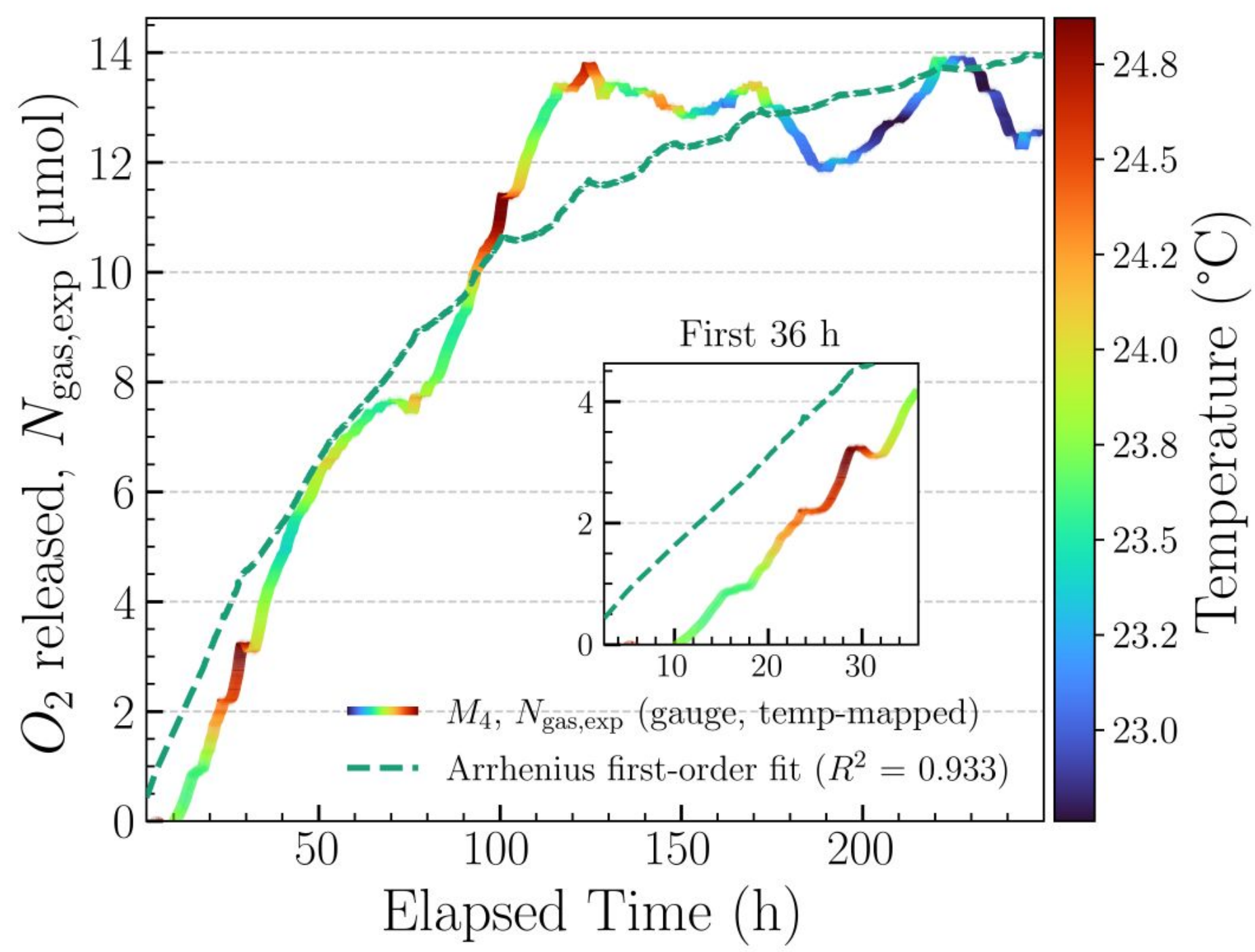

Figure S2: Oxygen release profile for sample M_4 (2.5 mM) measured at room temperature (RT) using gauge pressure sensing. The experimental gas-phase oxygen evolution, $N_{gas}(t)$, is shown as a function of elapsed time. Data illustrate slow-release kinetics under ambient conditions with temperature coded colormaps illustrating thermal drift effects characteristic of non-isothermal measurements.

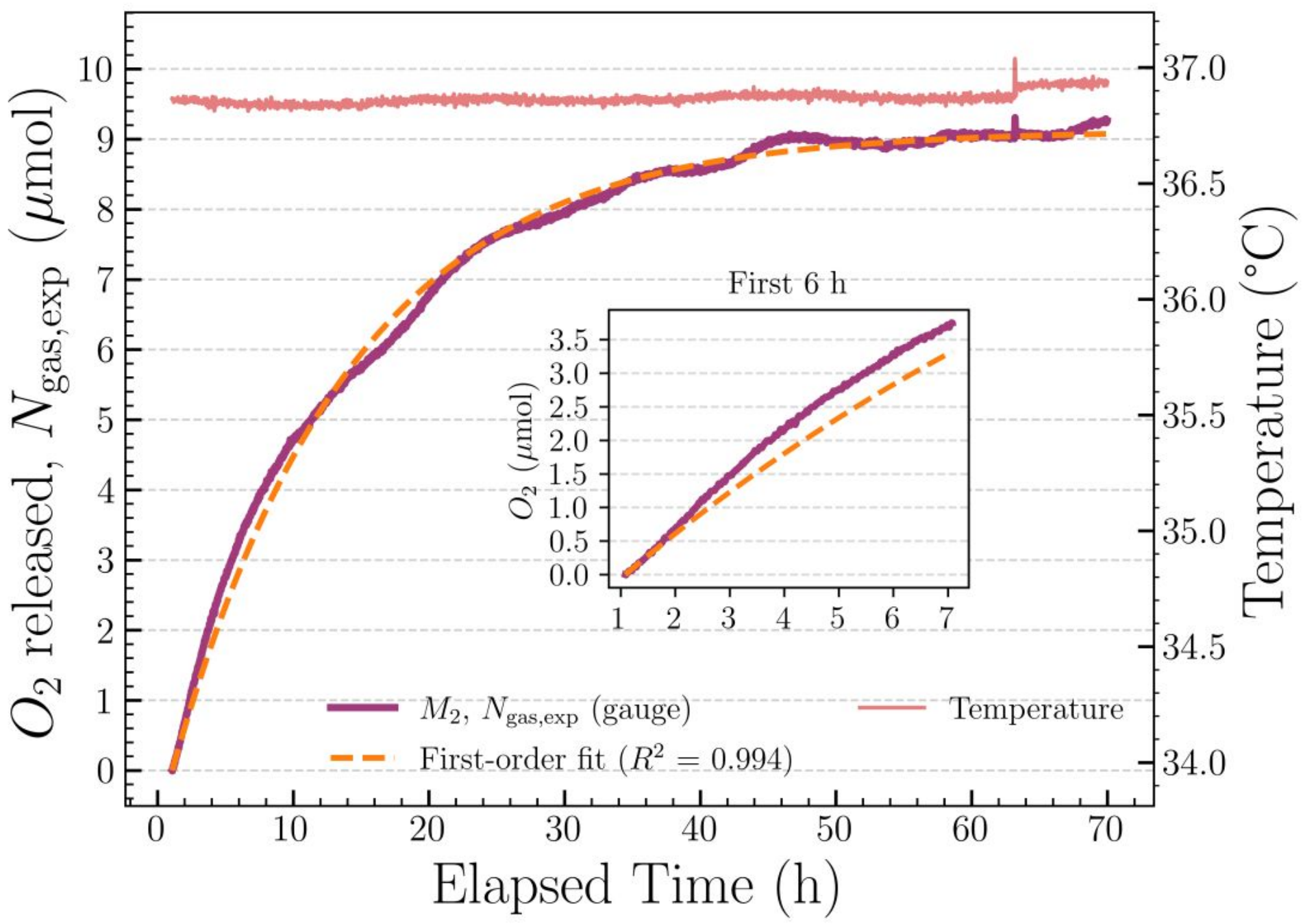

Figure S3: Oxygen release kinetics for sample M_2 (1.6 mM) at 37 °C measured using gauge pressure sensing. Experimental data are shown together with first-order kinetic fits, illustrating isothermal behavior and gas-phase recovery magnitude.

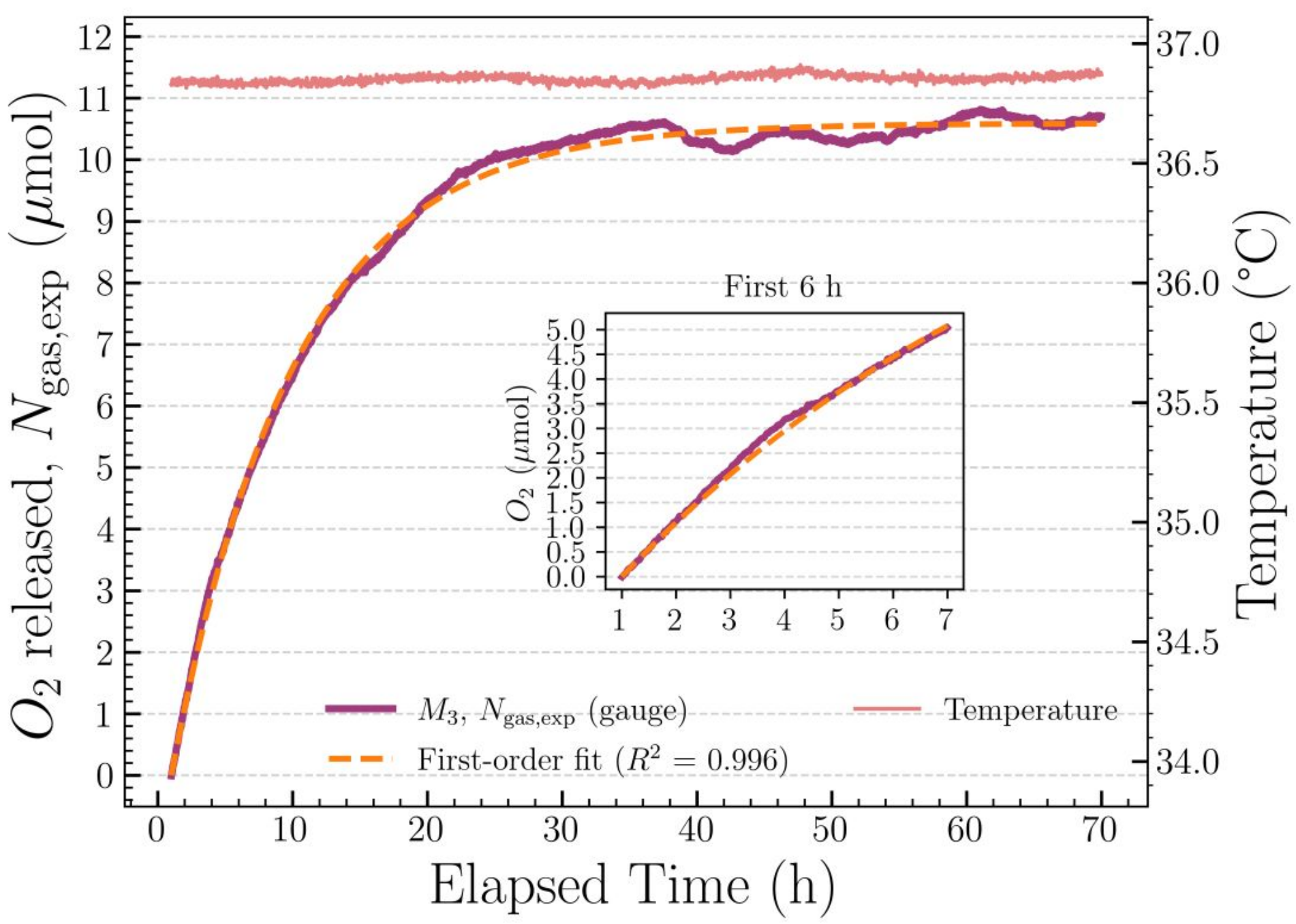

Figure S4: Oxygen release kinetics for sample M_3 (2.0 mM) at 37 °C measured using gauge pressure sensing. The fitted first-order model captures the initial release rate and the recovered gas-phase amplitude under controlled temperature conditions.

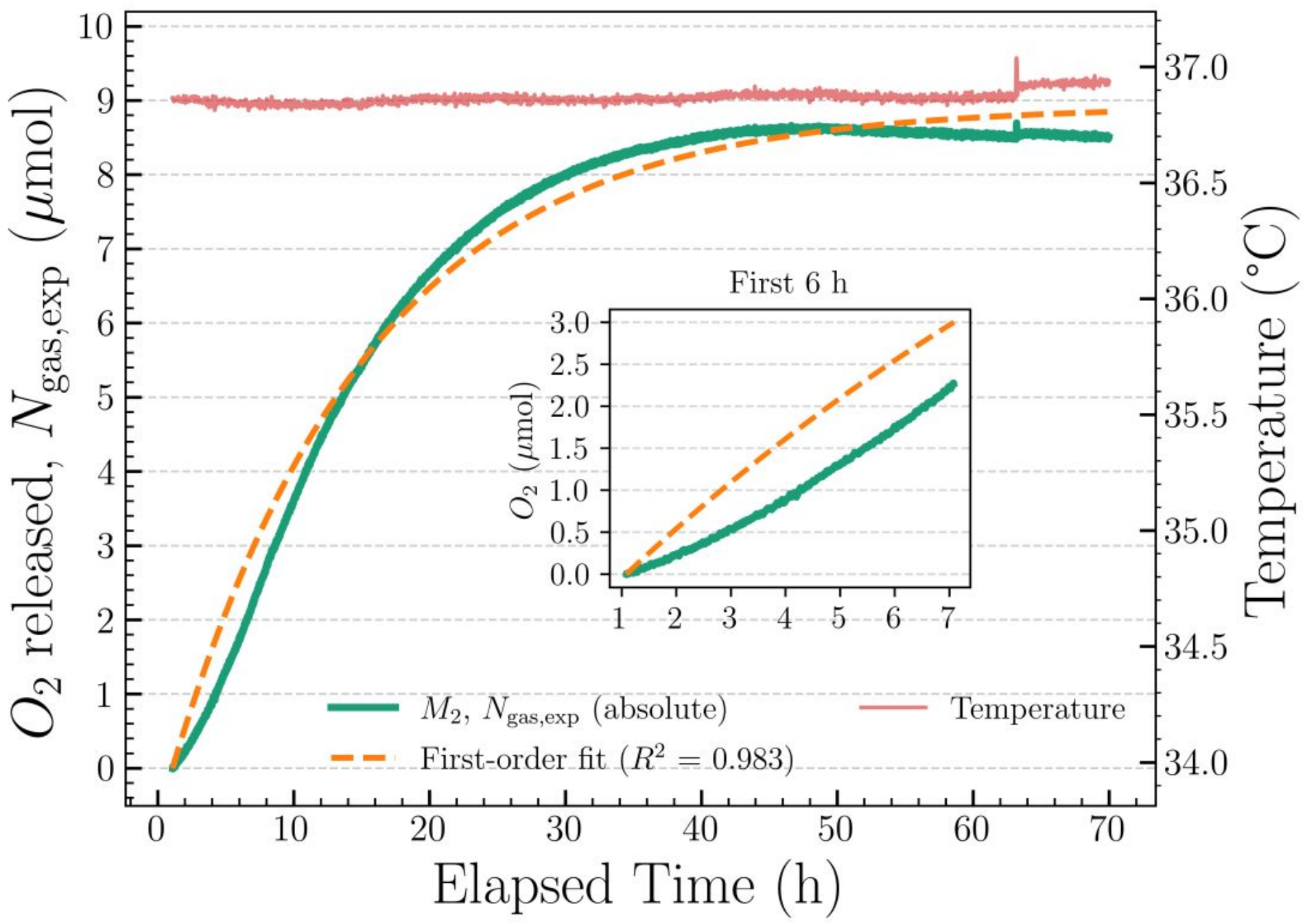

Figure S5: Oxygen release kinetics for sample M_2 (1.6 mM) at 37 °C measured using absolute pressure sensing. Consistent kinetic behavior is observed compared to gauge measurements, with systematic sensor-dependent offsets in absolute pressure conversion.

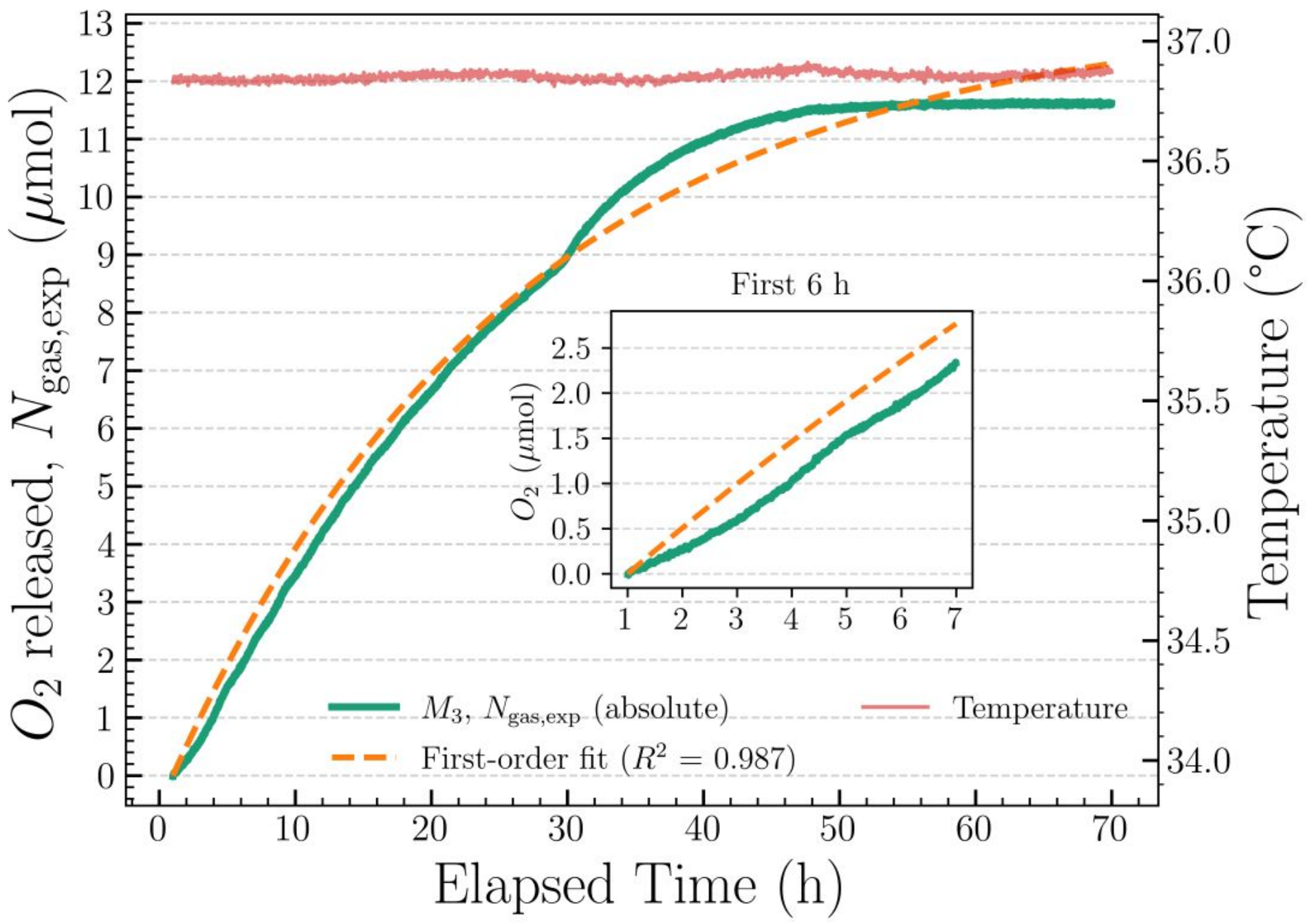

Figure S6: Oxygen release kinetics for sample M_3 (2.0 mM) at 37 °C measured using absolute pressure sensing, demonstrating reproducible first-order behavior under isothermal conditions.

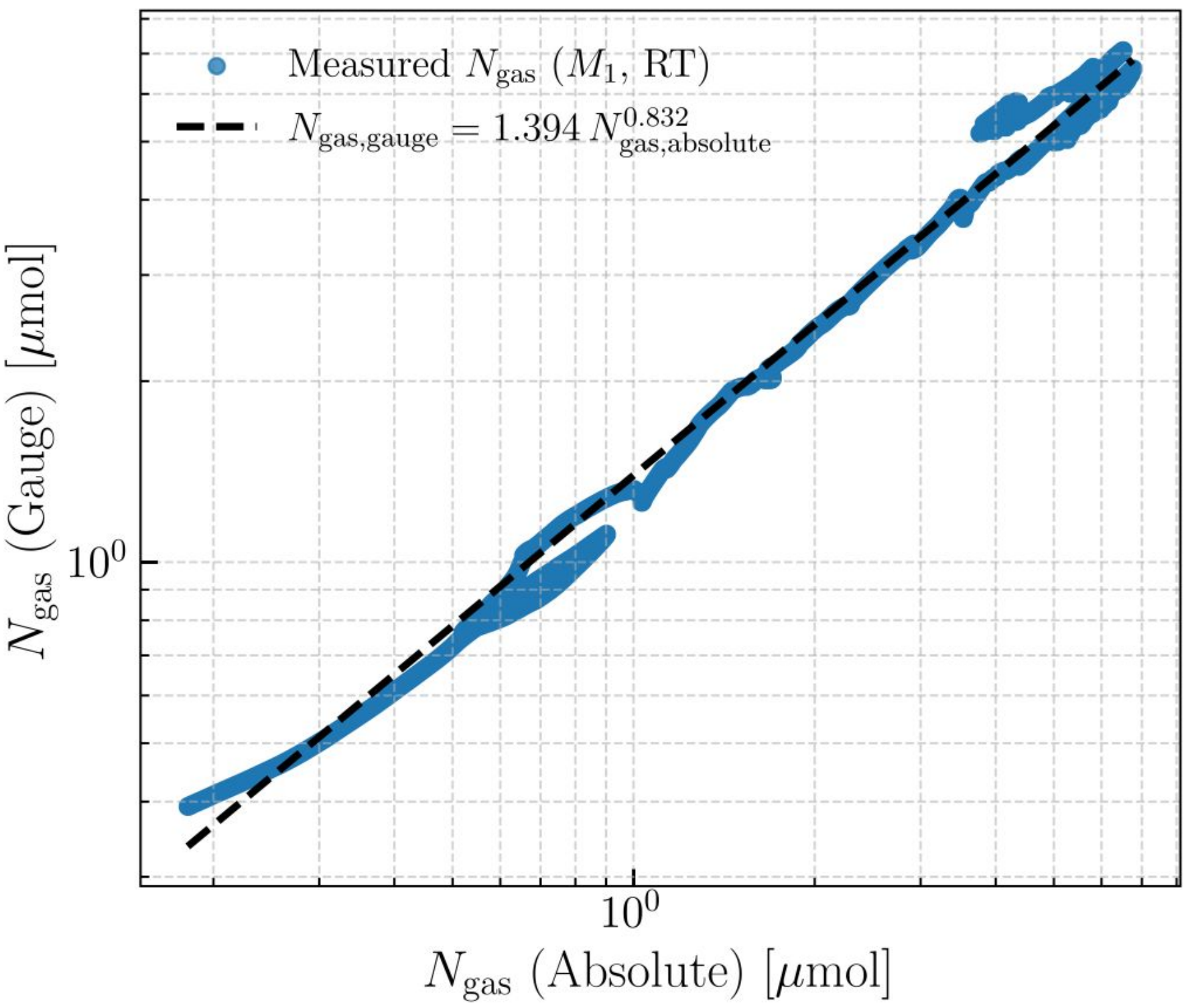

Figure S7: Log–log comparison of gas-phase oxygen release measured using gauge and absolute pressure sensing for sample M_1 (1.2 mM) at room temperature (RT). While absolute values differ due to sensor characteristics, both modalities capture comparable gas-phase accumulation scaling over the analysis window.

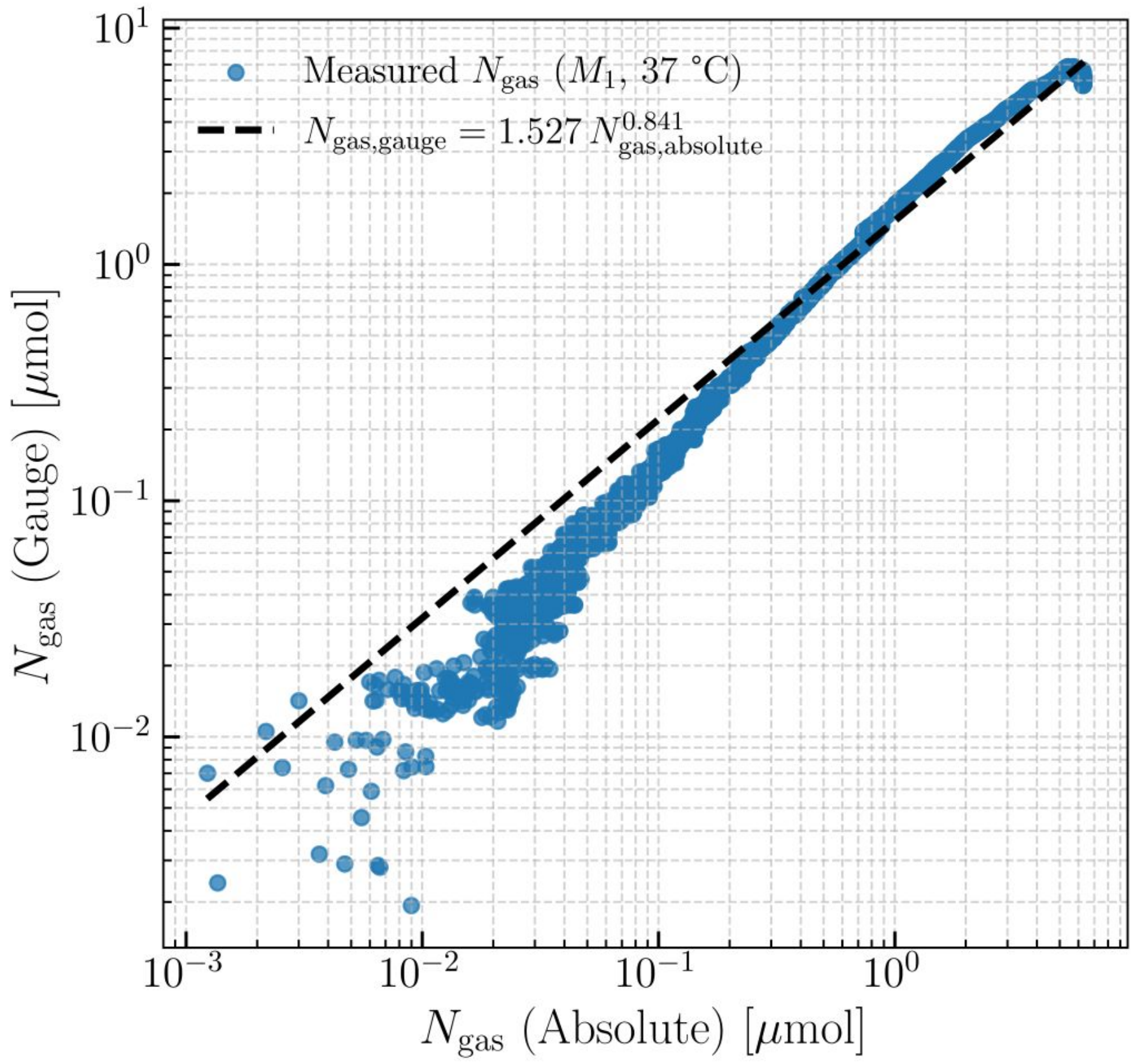

Figure S8: Log–log comparison of gauge and absolute sensing modalities for sample M_1 (1.2 mM) at 37 °C, highlighting consistent scaling behavior under isothermal conditions.

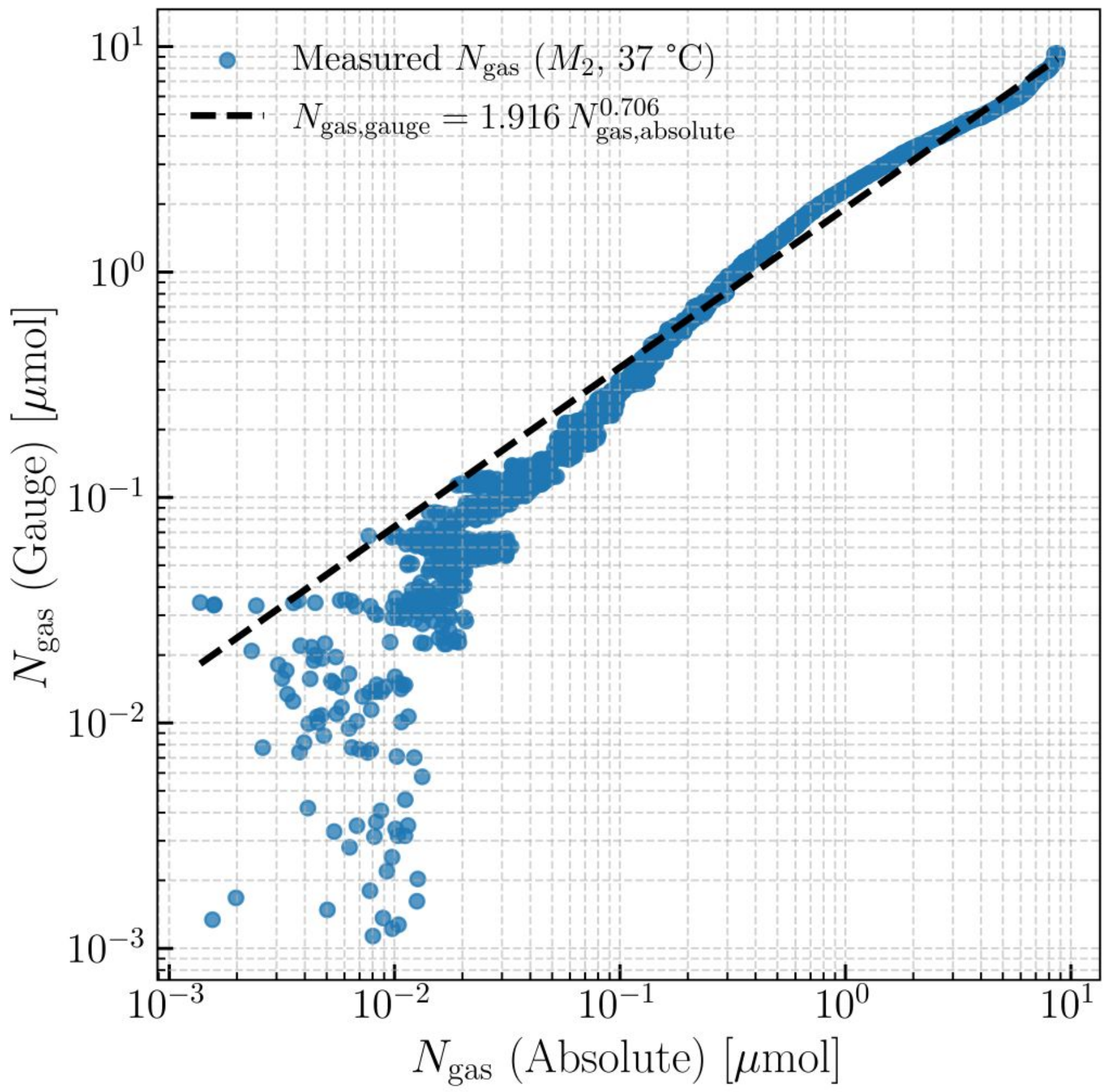

Figure S9: Log–log comparison of gauge and absolute sensing modalities for sample M_2 (1.6 mM) at 37 °C, highlighting consistent scaling behavior under isothermal conditions.

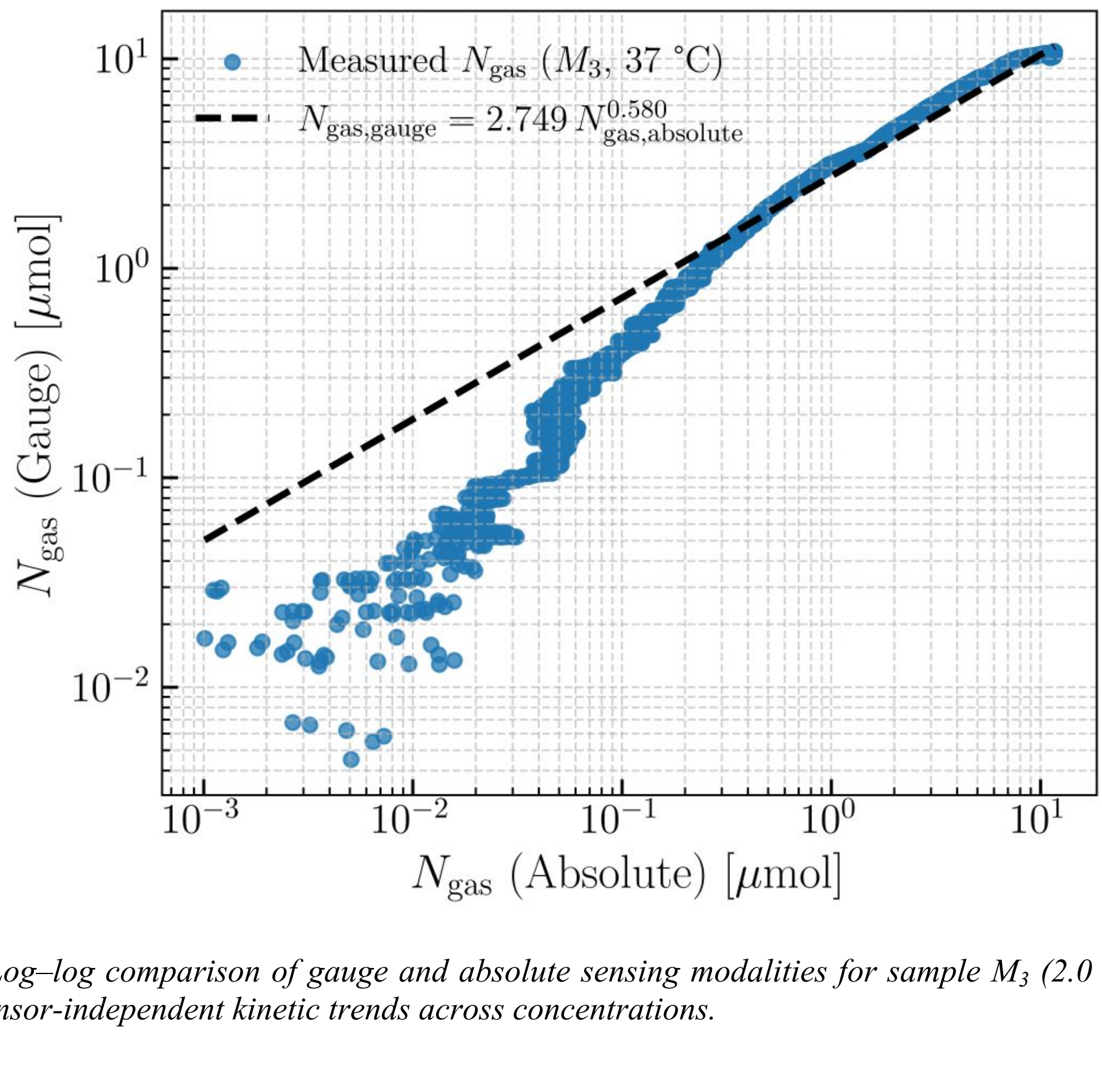

Figure S10: Log–log comparison of gauge and absolute sensing modalities for sample M_3 (2.0 mM) at 37 °C, confirming sensor-independent kinetic trends across concentrations.

1.2 Model Validation on Independent Dataset

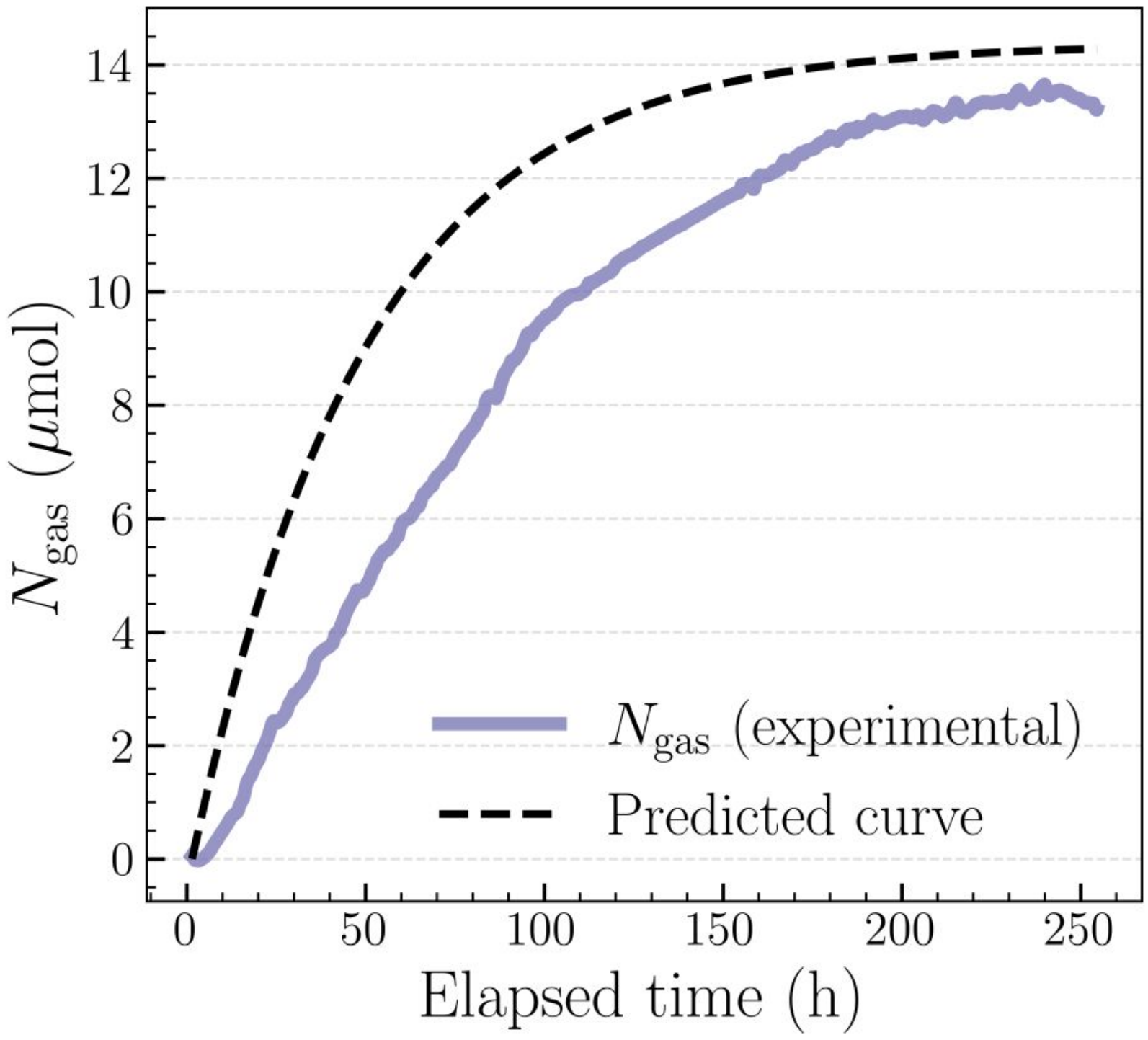

Figure S11: Model validation for independent dataset (2.67 mM) at room temperature (RT) using absolute pressure sensing. Experimental oxygen release was compared against model predictions derived from pooled parameters obtained from M_1–M_4. Deviations in kinetic rate reflect temperature mismatch relative to the isothermal training data.

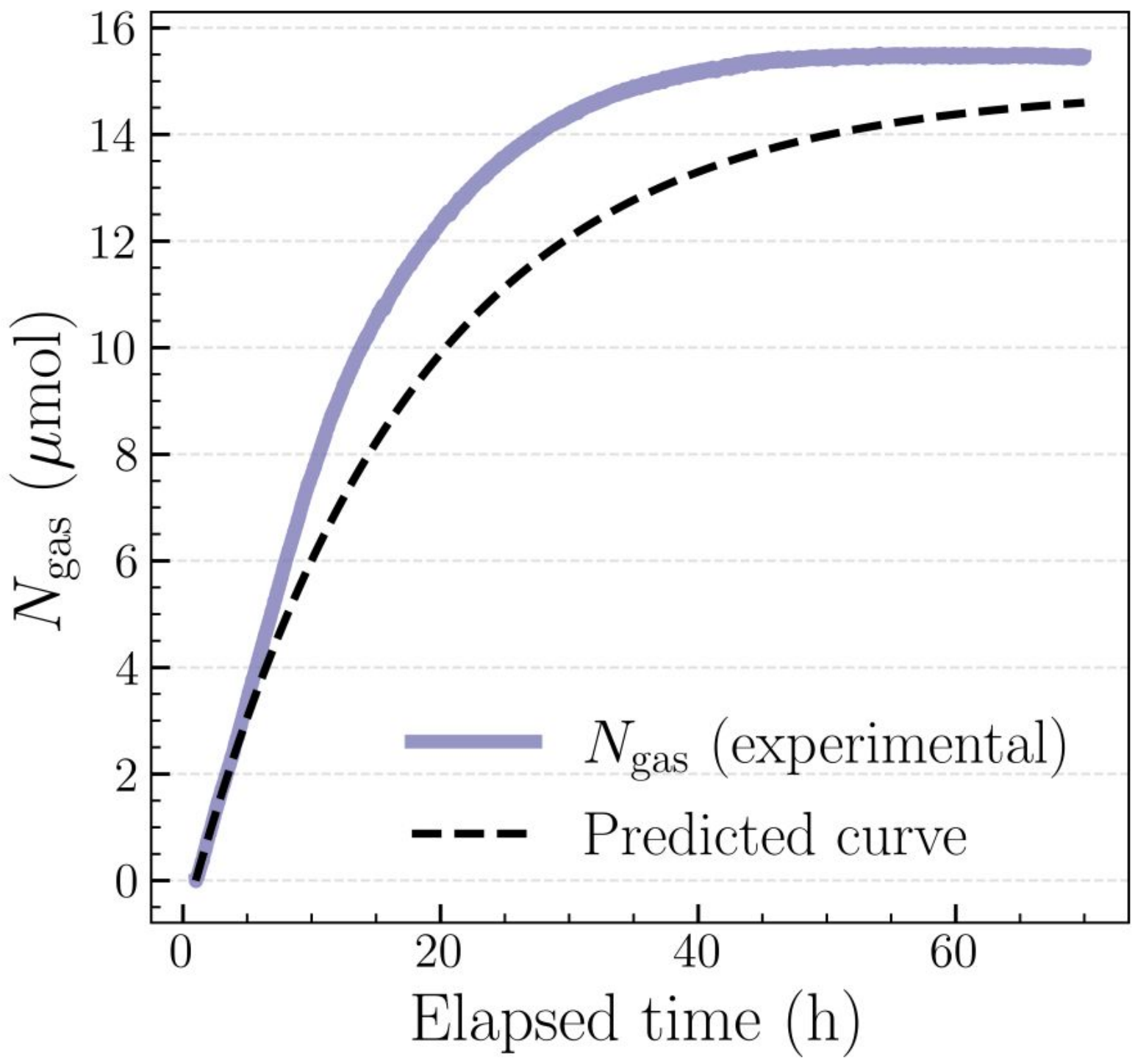

Figure S12: Model validation for independent dataset (2.67 mM) at 37 °C using absolute pressure sensing. The model accurately reproduces both the temporal evolution and gas-phase recovery magnitude without refitting, demonstrating predictive capability under matched temperature conditions.

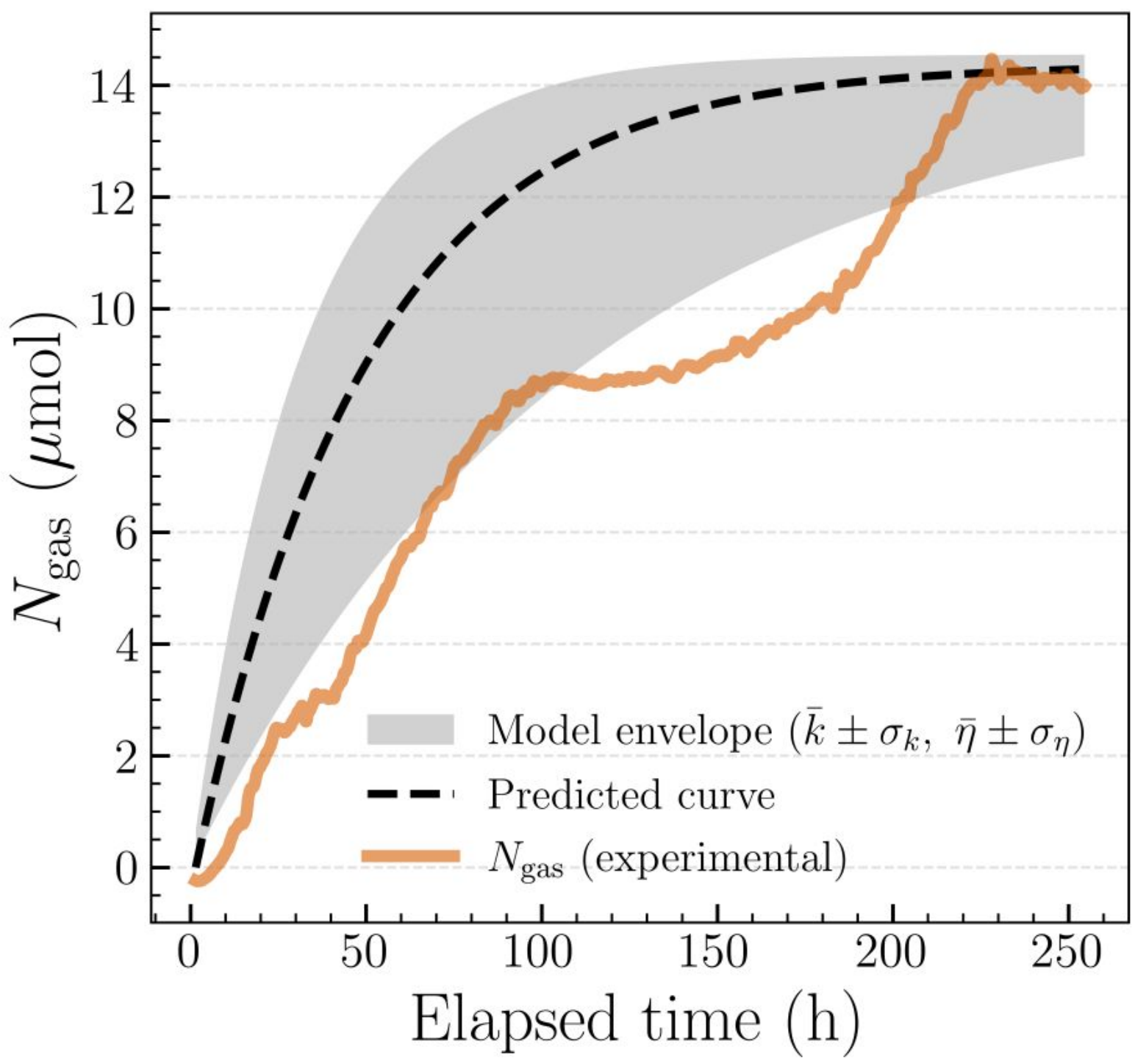

Figure S13: Uncertainty propagation of model predictions for dataset (2.67 mM) at room temperature (RT) using gauge pressure sensing. The shaded envelope represents parameter variation arising from (k(mean) ± σ) and (η(mean) ± σ).

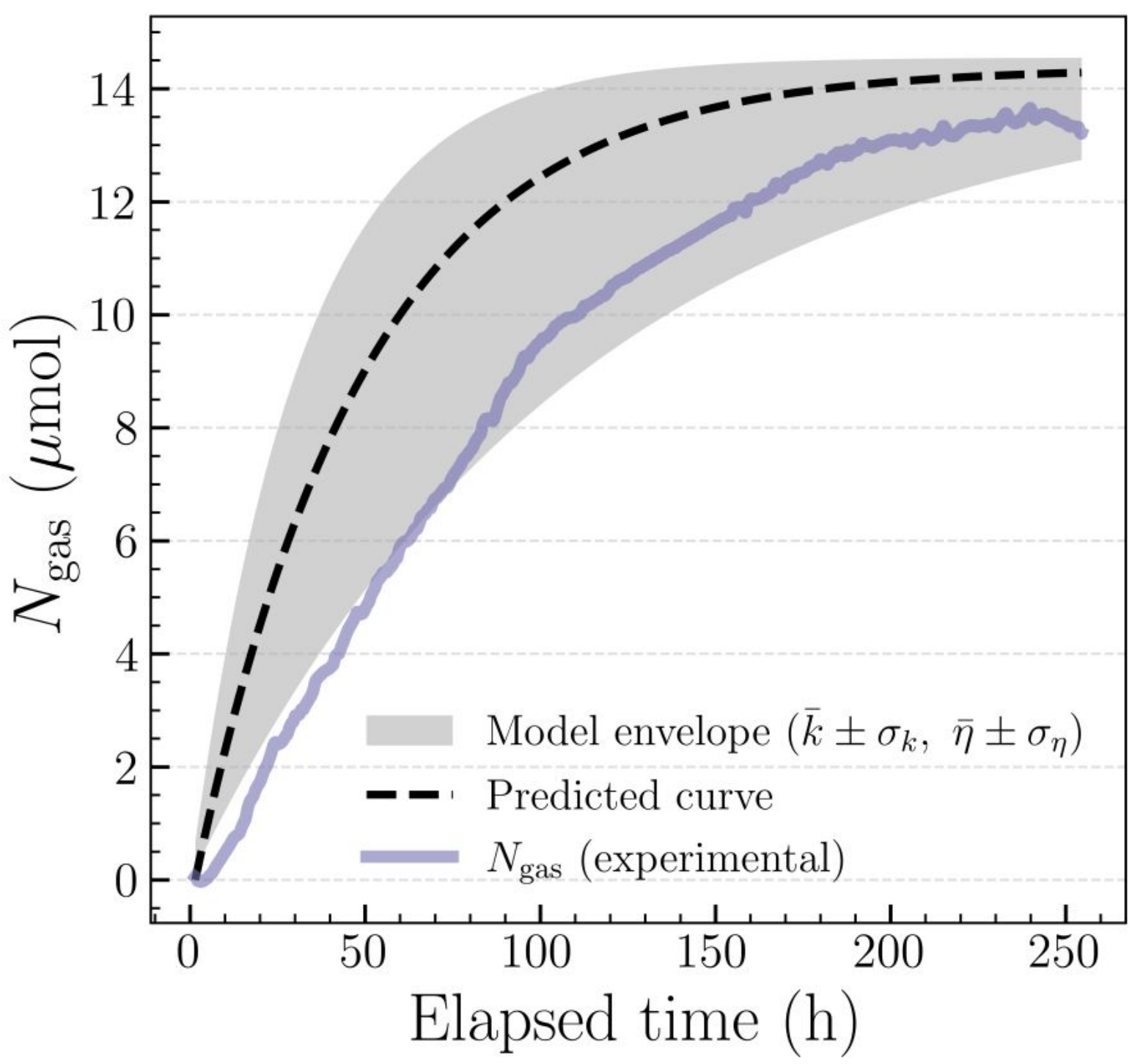

Figure S14: Uncertainty propagation of model predictions for dataset (2.67 mM) at room temperature (RT) using absolute pressure sensing.

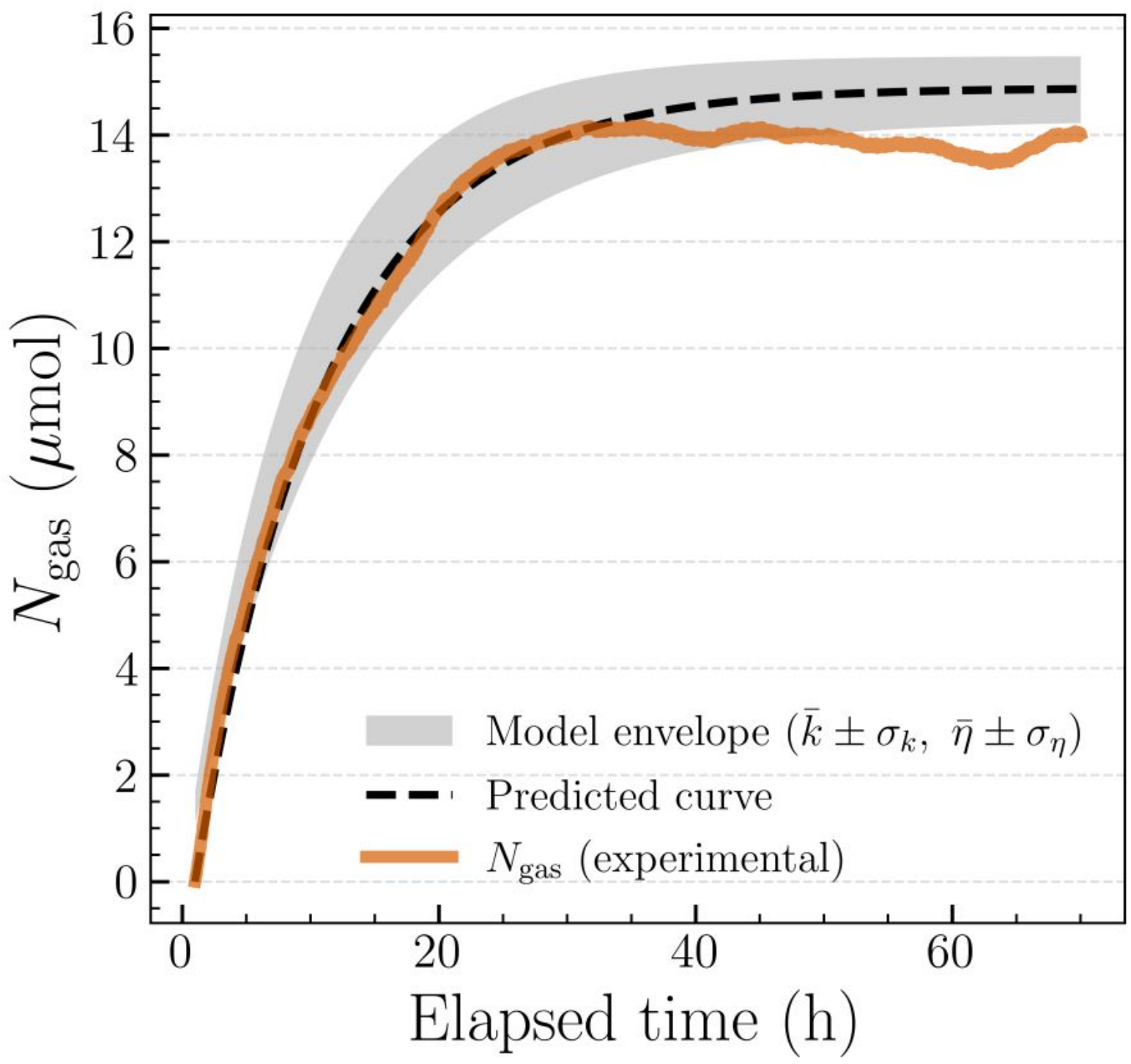

Figure S15: Uncertainty propagation of model predictions for dataset (2.67 mM) at 37 °C using gauge pressure sensing. Experimental data fall within the predicted envelope, indicating robust parameter transferability under isothermal conditions.

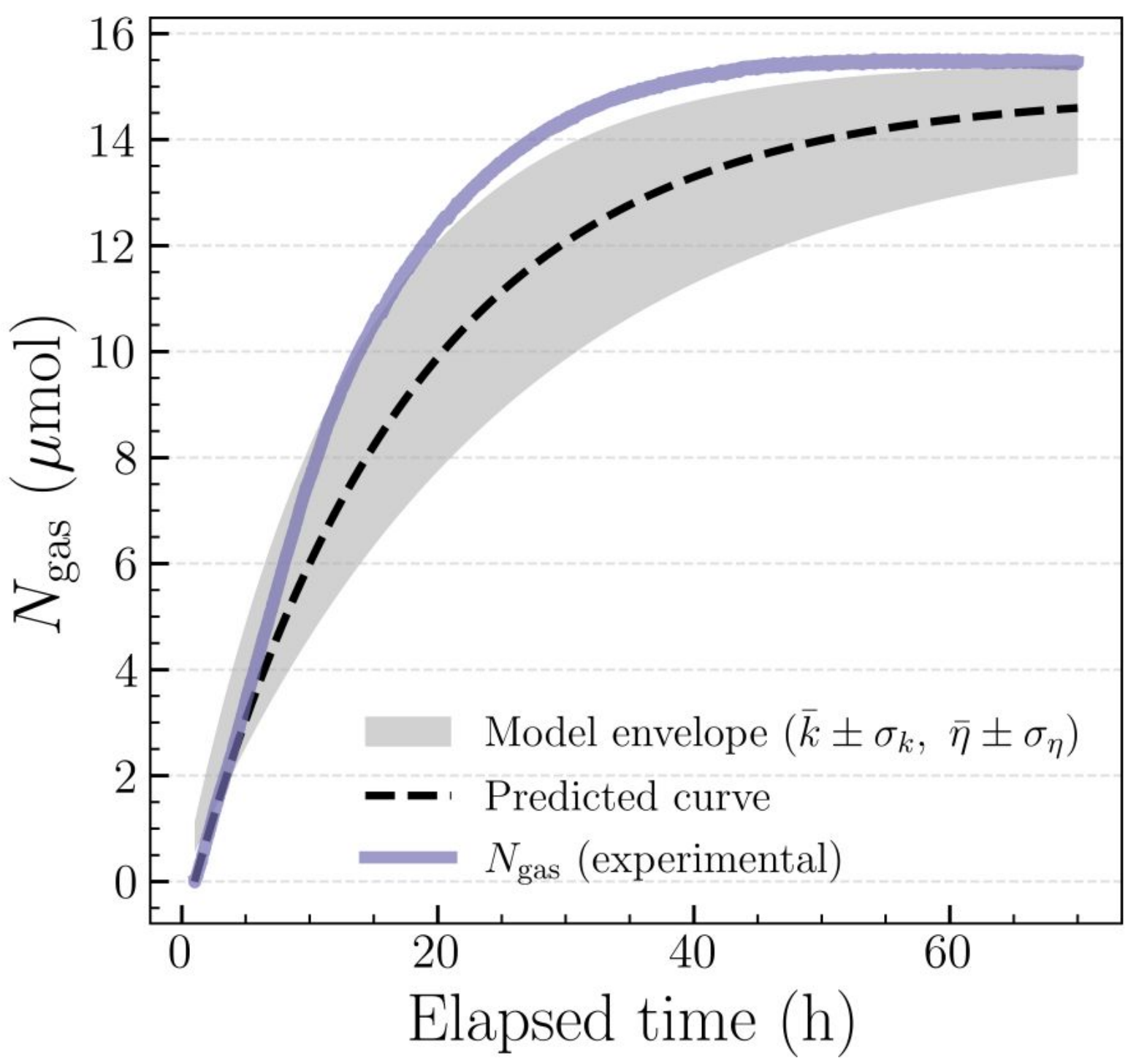

Figure S16: Uncertainty propagation of model predictions for dataset (2.67 mM) at 37 °C using absolute pressure sensing.

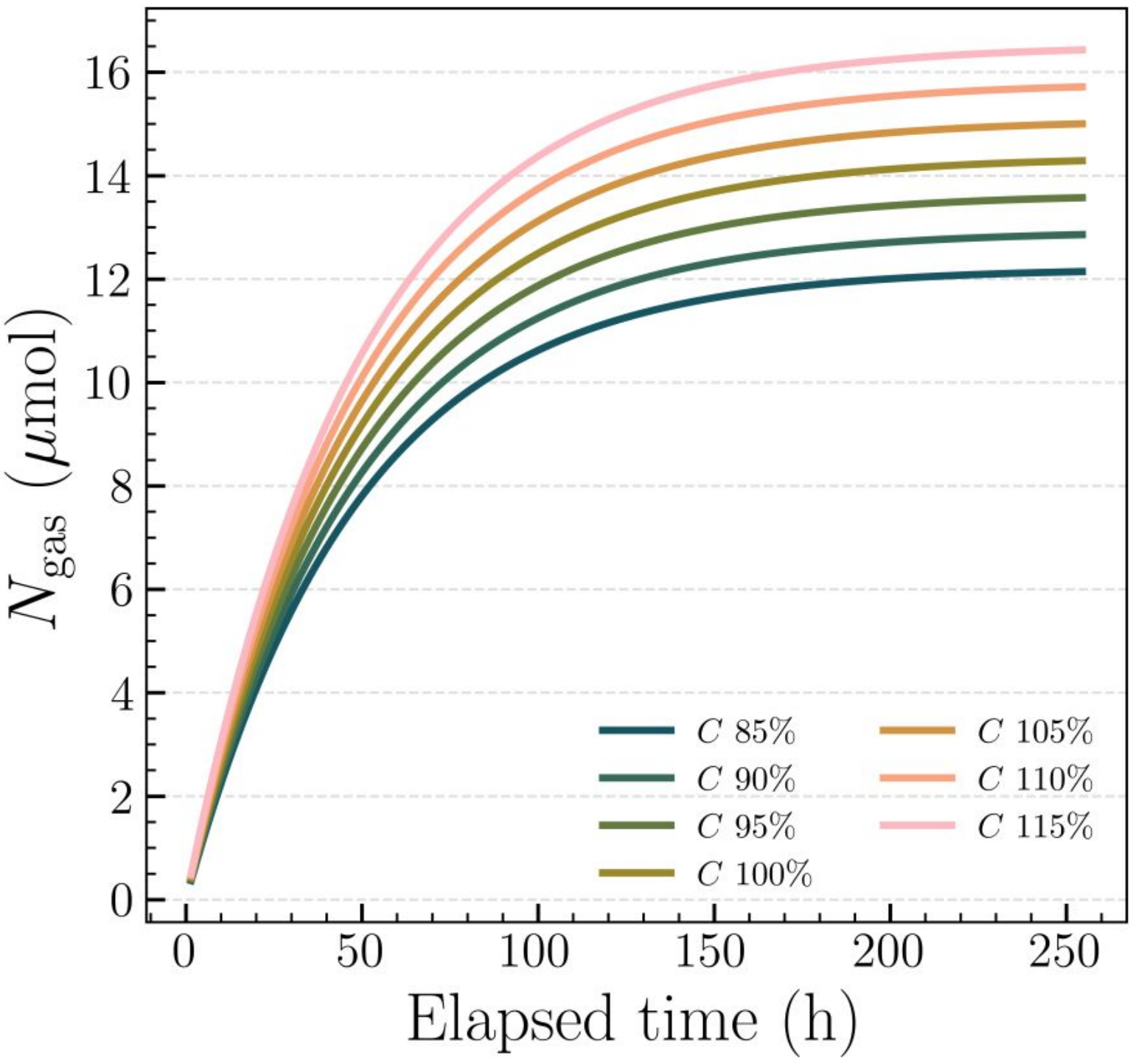

Figure S17: Sensitivity of predicted oxygen release profiles to concentration variations (±15%) at room temperature (RT) for gauge pressure sensing, illustrating linear scaling of the recovered gas-phase oxygen with concentration.

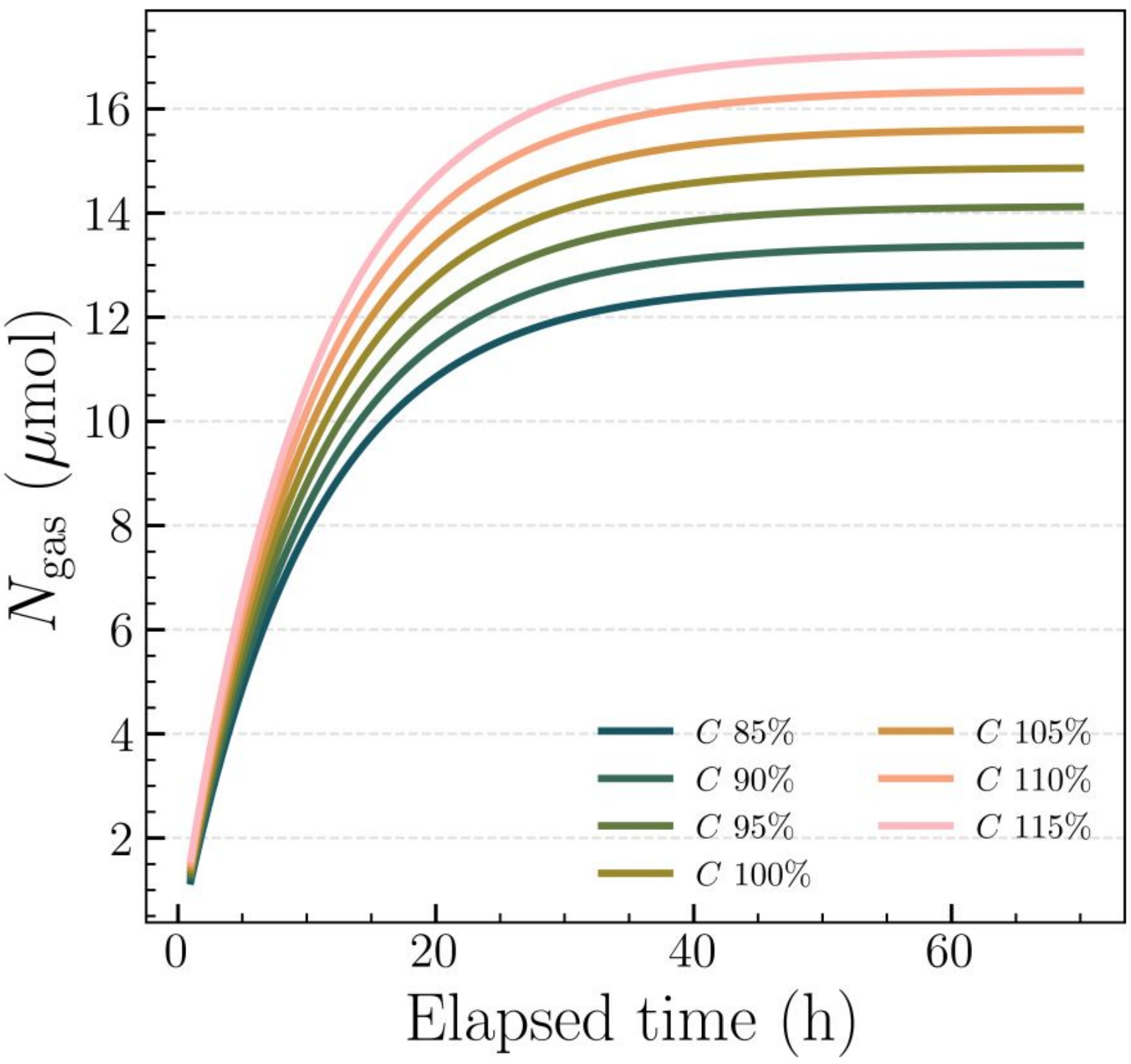

Figure S18: Sensitivity of predicted oxygen release profiles to concentration variations (±15%) at 37 °C for gauge pressure sensing. While recovered gas-phase magnitude scales with concentration, normalized release kinetics remain invariant.

Table S1: Summary of temperature statistics for all ANT-EPO measurements at room temperature (RT). Mean temperature, standard deviation (SD), and minimum/maximum values were calculated over the full measurement duration for each dataset. For room-temperature (RT) experiments, these values quantify ambient thermal variability accounted for via Arrhenius-based kinetic fitting.

Sample	[ANT-EPO] mM	Temp.	Range	Mean temp. (°C)	SD	Min T(°C)	Max T (°C)
M_1	1.2			27.15	0.99	23.96	29.66
M_2	1.6	RT	> 200 hours	23.08	0.43	22.35	24.18
M_4	2.5			23.81	0.43	22.96	25.41

Table S2: Effective rate constants (k_{exp}) extracted from pressure-derived gas-phase oxygen accumulation profiles as a function of ANT-EPO concentrations under ambient and isothermal conditions. At room temperature (RT), where ambient thermal drift was present, k values were reported as mean ± standard deviation derived from temperature-resolved Arrhenius modeling. Under isothermal conditions at 37 °C, a single characteristic rate constant was obtained per measurement.

Sample	Temperature (°C)	Sensor type	Effective rate constant k (h^{-1})	SD (h^{-1})	Remark
M1	RT	gauge	0.0330	0.0030	Arrhenius-based fit using time-resolved temperature
M2	RT	gauge	0.0159	0.0009	
M4	RT	gauge	0.0122	0.0008	
M1	37	gauge	0.09818	-	Stable temperature
		absolute	0.04689	-	
M2	37	gauge	0.07972	-	
		absolute	0.07874	-	
M3	37	gauge	0.11477	-	
		absolute	0.04671	-	

Table S3: Apparent gas-phase recovery factors, η, used to scale the theoretical oxygen capacity to the pressure-derived gas-phase oxygen amount within the analysis window.

Sample	Temperature (°C)	Sensor type	Apparent gas-phase recovery factor, η (%)
M1	RT	gauge	94.14
M2	RT	gauge	94.25
M4	RT	gauge	91.80
M1	RT	absolute	93.00
M1	37	gauge	95.40
		absolute	88.00
M2	37	gauge	96.00
		absolute	90.60
M3	37	gauge	90.10
		absolute	97.10

Table S4: Comparison of experimental and model-predicted characteristic release times t_{25} and t_{50} for validation dataset 2.67 mM at 37 °C. Characteristic times are defined as the time required to reach 25% and 50% of the final recovered gas-phase oxygen amount, respectively.

Sensor modality	Temp.	Time	Experimental (h)	Predicted (h)
Gauge	37 °C	t_{25}	3.21	3.95
		t_{50}	7.16	8.1
Absolute		t_{25}	5.54	5.9
		t_{50}	10.17	12.7

Table S5: Model validation metrics for dataset 2.67 mM at room temperature (RT), including root mean square error (RMSE) and relative error in the recovered gas-phase model, N_0.

Sensing modality	RMSE (μmol)	Relative error (%)
Gauge	3.56	− 2.8
Absolute	2.58	− 8.1

Table S6: Model validation metrics for dataset 2.67 mM at 37 °C. In addition to RMSE and relative error in recovered gas-phase model magnitude, N_0, normalized RMSE was reported to enable scale-independent comparison.

Sensing modality	RMSE (μmol)	Relative error (%)	Norm. RMSE (%)
Gauge	0.66	− 5.8	4.7
Absolute	1.75	+ 3.9	11.3

Table S7: Pearson correlation coefficients, r, between experimental and model-predicted oxygen release trajectories for validation dataset. Correlation was evaluated over the full temporal evolution of $N_{gas}(t)$.

Sensing modality	Temperature	Pearson coefficient, r
Gauge	RT	0.917
Absolute		0.964
Gauge	37 °C	0.992
Absolute		0.987

1.3 Validation Metrics

1.3.1 Relative error in gas-phase recovery magnitude

The relative error in the recovered gas-phase model amplitude N_0 was calculated as:

$$Relative\ error\ (\%) = \frac{N_{0,exp} - N_{0,model}}{N_{0,model}} \times 100$$

1.3.2 Pearson correlation coefficient

The Pearson correlation coefficient, *r,* between experimental and predicted oxygen release trajectories was calculated as:

$$r = \frac{\sum_i \left(N_{exp,i} - \overline{N}_{exp}\right)\left(N_{pred,i} - \overline{N}_{pred}\right)}{\sqrt{\sum_i \left(N_{exp,i} - \overline{N}_{exp}\right)^2}\sqrt{\sum_i \left(N_{pred,i} - \overline{N}_{pred}\right)^2}}$$

where the summation runs over all measured time points. Pearson correlation was used to assess similarity in temporal evolution independent of absolute magnitude.
